# The interplay between hydraulic capacitance and stomatal regulation strategy affects soil–plant hydraulics and transpiration

**DOI:** 10.1111/nph.71143

**Published:** 2026-03-30

**Authors:** Stefano Martinetti, Andrea Carminati, Peter Molnar, Marius G. Floriancic

**Affiliations:** ^1^ Department of Civil, Environmental & Geomatic Engineering ETH Zurich Zurich 8093 Switzerland; ^2^ Department of Environmental System Sciences ETH Zurich Zurich 8092 Switzerland

**Keywords:** beech, hydraulic capacitance, leaf water potential, plant water storage, soil–plant hydraulics, spruce, stomatal regulation strategy, transpiration

## Abstract

- Plant water storage contributes to transpiration, but it is unclear how its relevance in supporting transpiration depends on the stringency of stomatal regulation.
- Here, we show the compounding effect of stomatal regulation and hydraulic capacitance on plant water use, by means of a soil–plant hydraulic model and measurements of leaf water potential, sap flow, stomatal conductance and capacitance in beech and spruce in the field.
- We found that large capacitance led to a large buffering effect on leaf water potential, explained by increasing amounts of transpiration sourced from internal plant water storage. However, the extent to which capacitance allows plants to sustain transpiration depends on the stringency of stomatal regulation. For stomata that limit leaf water potential at a fixed threshold (as observed in spruce), large capacitance increased transpiration throughout all soil water conditions. By contrast, for flexible stomatal regulation mechanisms optimizing transpiration over leaf water potential (as observed in beech), large capacitance caused stomata to close earlier in the day under wet soil conditions.
- Our findings suggest a trade‐off between developing tissues that can store large water volumes and stomatal regulation mechanisms that allow leaf water potential to reach more negative values during periods of high transpiration demand.

## Introduction

Plants take up water from the soil to meet their transpiration demand. However, significant amounts of water are retained in plant tissues (from here on called ‘plant water storage’), and water can be accessed from these storages at sufficiently negative water potential. Plant water storage capacity and the relationship between plant water storage and water potential (from here on called ‘capacitance’) has been a growing research topic in recent years.

Vegetation in arid ecosystems has been shown to rely on plant water storage for transpiration (e.g. Preisler *et al*., 2022; Wang *et al*., 2024), but plant water storage and capacitance have also been shown to affect vegetation functioning in humid ecosystems (e.g. Gleason *et al*., 2014; Richards *et al*., 2014; Matheny *et al*., 2015). Water movement from plant water storage into the transpiration stream might buffer imbalances between water supply and demand (Meinzer *et al*., 2001; Fuchs, 2025) allowing leaf water potential to remain at levels that plants typically operate at. Also, by shifting the water source for transpiration from the soil (i.e. by root water uptake) to plant water storage, the timing of daily peak sap flow might lag behind the timing of daily peak transpiration. Furthermore, the timing of daily peak root water uptake might lag behind the timing of daily peak sap flow, depending on where within a tree water is stored (Kramer, 1937; Mcculloh *et al*., 2014). The hypothesis that plant water storage affects timing and magnitude of water fluxes, thereby causing a characteristic time lag between peak water fluxes across the soil–plant‐atmosphere continuum has previously been tested in various ecosystems (Burgess & Dawson, 2008; Phillips *et al*., 2009). For example, time lags in pine trees were roughly 50 min for Loblolly pine, but up to 5 h for Aleppo pine under drought conditions (Phillips *et al*., 1997; Preisler *et al*., 2022). The time lag is expected to grow with increasing capacitance and plant water storage; thus, large trees exhibit larger time lags than grasses (Feldman *et al*., 2021).

Plant water storage capacity is particularly large in sapwood (Waring & Running, 1978), and sapwood capacitance directly governs water relations of the entire tree (Meinzer *et al*., 2003, 2006). Previous studies have linked ratios between heartwood and sapwood area to drought vulnerability, as species with larger sapwood area exhibit higher drought tolerance (Matusick *et al*., 2022). Plant species show high variability in plant water storage capacity and capacitance (Goldstein *et al*., 1984), and individual beech and spruce trees have shown variable stem water storage across seasons and under drought stress (Knüver *et al*., 2022). However, it is not clear how varying storage capacity and hydraulic capacitance interact with stomatal regulation.

Stomatal regulation strategies have been compared across species based on leaf water potential dynamics, thereby creating a ‘spectrum’ of strategies (e.g. Stocker, 1956; Jones, 1998; Tardieu & Simonneau, 1998; Meinzer *et al*., 2017). At the limits of this spectrum, species defined as ‘strict’ isohydric keep leaf water potential above a constant value as environmental conditions change and soil water potential drops, while species defined as ‘strict’ anisohydric allow leaf water potential to become more negative with soil drying and dropping soil water potential. The isohydric/anisohydric spectrum can be useful to show coordination between stomatal regulation strategy (degree of iso/anisohydricity) and some plant hydraulic traits (e.g. xylem hydraulic conductance, Sperry, 2004), and studies have shown trade‐offs between capacitance, embolism resistance and transpiration (Gleason *et al*., 2014). Capacitance has been included in soil–plant hydraulic and even terrestrial ecosystem flux models (Hunt & Nobel, 1987; Carlson & Lynn, 1991; Kaner *et al*., 2020) and has also been linked to carbon assimilation (Hartzell *et al*., 2017). However, a potential soil–plant hydraulic feedback between capacitance and stomatal regulation strategies has not been quantified yet to our knowledge, although different stomatal regulation strategies and varying capacitance are observed among species.

In this study, we hypothesize that hydraulic capacitance affects transpiration differently depending on the stomatal regulation strategy. We aim to address the interaction between stomatal regulation strategy and degree of capacitance with experimental data and simulations from a soil–plant hydraulic model (Carminati & Javaux, 2020). First, we present experimental data of beech and spruce trees from the ‘WaldLab’ field site in Zurich, showing contrasting stomatal regulation strategies and different degrees of capacitance. Then we use the model in order to explore the interaction between stomatal regulation strategy and hydraulic capacitance under varying soil water potential. For this purpose, we extended the model with a capacitance module. We performed soil–plant hydraulic simulations of trees employing a ‘strict’/isohydric stomatal regulation and a ‘flexible’/anisohydric stomatal regulation strategy with variable plant water storage capacity and capacitance. Our approach gives insight into the interaction of plant–hydraulic traits and the development of sustainable soil–plant hydraulic strategies in the context of species survival under drought conditions.

## Materials and Methods

### Field site and measurement setup

**Fig. 1:**
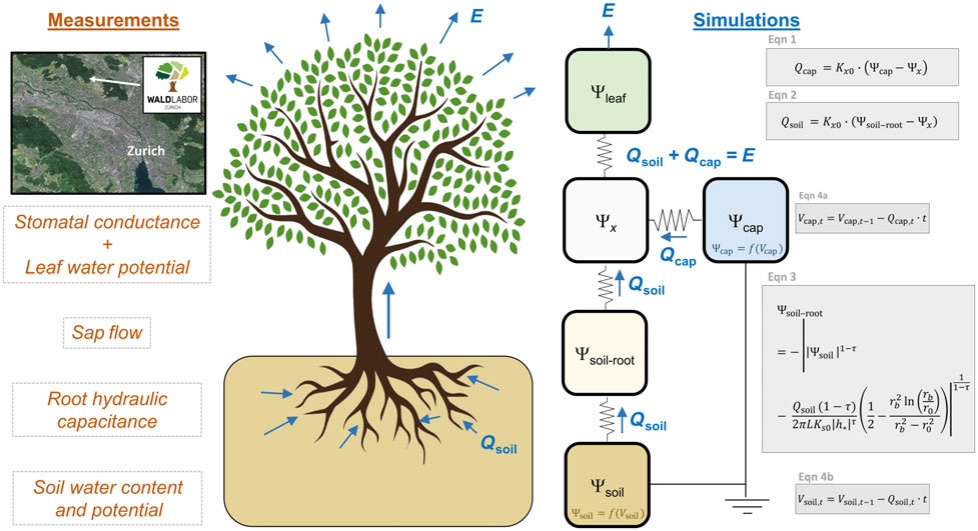
Location of the ‘WaldLab Forest Experimental Site’ in Zurich (top left corner) and a conceptual diagram of the performed measurements (left). Scheme of the extended soil–plant hydraulic model used in this study (right), with symbols defined in Table 1, Supporting Information Table S1 and Methods S4. Measured sap flow at breast height integrates water fluxes from plant and soil water storage at breast height but lacks information of water fluxes from plant water storage further up the transpiration stream. In the model, transpiration *E* at each time step is equal to the sum of water fluxes coming from root water uptake *Q*_soil_ and plant water storage *Q*_cap_. The resistances across the soil–plant hydraulic compartments are not equal: Between the soil and the soil–root interface, the resistance is equal to the soil hydraulic resistance for the given soil water potential Ѱ_soil_, whereas the other resistances along the tree were set equal to an overall soil–plant hydraulic resistance as estimated from data (see ‘Model parameterization, calibration and validation’ in the Materials and Methods section for details). At the onset of transpiration, Ѱ_soil_ and Ѱ_cap_ are assumed to be in equilibrium, and Ѱ_cap_ is obtained from the water storage – potential relationship characterizing capacitance (*C* = d*V*_cap_/dѰ_cap_ and Eqns S5 and S6), whereas Ѱ_soil_ is obtained from the soil water retention curve (Fig. S2). Methods S4 contain a detailed model description.

We conducted measurements of soil–plant hydraulic dynamics at the ‘WaldLab Forest Experimental Site’ Zurich in Switzerland (see Fig. 1 and Supporting Information Fig. S1). It is a typical central European managed mixed beech (*Fagus sylvatica* L.) and spruce (*Picea abies* (L.) H. Karst) forest, situated on a south‐west facing slope. Data collected between 2010 and 2022 have a mean annual temperature of 9.3°C and mean annual precipitation of 1134 mm. Climatic variables were measured with an Atmos 41 all‐in‐one weather station (Meter Group GmbH, München, Germany) located *c*. 150 m outside of our forest plot. The soil at the site is *c*. 1 m deep covering moraine material. Soil water contents and soil water potentials were measured every 10 min at 10, 20, 40 and 80 cm depth with Teros 11/12 sensors (Meter Group AG) and Teros 21/32 sensors (Meter Group GmbH), respectively, and were conducted close to the studied beech and spruce trees, respectively (Fig. S1).

During the growing seasons 2021, 2022 and 2023, we conducted manual measurements of stomatal conductance with an SC1 leaf porometer (Meter Group GmbH) and leaf water potential with a Scholander bomb (Soil Moisture Inc., Goleta, CA, USA) around midday (at *c*. 11:00 h CET) on two beech and two spruce trees. Measurement campaigns were carried out throughout the main growing seasons of 2021 (nine campaigns) and 2023 (nine campaigns), and more extensively during the main growing season 2022 (40 campaigns). We conducted at least two measurements per tree on sun‐exposed leaves for both stomatal conductance and leaf water potential and averaged the two measurements. In addition, we measured stomatal conductance and leaf water potential every 2 h, typically from sunrise to sunset, on 7 d during the main growing season of 2022. In total, we collected 217 measurements on beech trees and 189 measurements on spruce trees, of both stomatal conductance and leaf water potential. During the campaigns, we also measured relative humidity and temperature at the respective leaves manually, with a SHT85 sensor (Sensirion AG, Staefa, Switzerland) to account for microclimatic effects at the leaf scale. Due to the lack of radiation protection, these measurements are prone to errors from sensor overheating, thus local VPD estimates were corrected with VPD measurements taken at the nearby weather station (further described in the Methods S1). One beech and one spruce tree were equipped with sap flow sensors (TreeTalker, Nature 4.0, Viterbo, Italy) to measure sap flow density at breast height at hourly resolution by the heat dissipation method (Granier 1985), calibrated with species‐specific parameter values (Methods S2). Another beech and another spruce tree were equipped with SFS2 sap flow sensors (UP GmbH, Stendal, Germany) measuring sap flow density at 10‐min resolution at breast height. The SFS2 sensors measured at higher temporal resolution, provided more consistent subdaily dynamics and were therefore more suitable to assess soil–plant hydraulics at high temporal resolution. We compared the timing of maximum daily sap flow (assessed based on readings from SFS2 sensors) to the timing of maximum daily root water uptake, which we estimated from soil water content sensors at 10, 20, 40 and 80 cm installed close to the beech and spruce trees. We computed the time derivative to translate soil water content to water fluxes, applied a 2‐h time window moving average twice to smoothen the signal and selected the timing of the daily minimum (i.e. when the rate of decreasing water content was highest) as the timing of maximum root water uptake. For the analysis we omitted wet days, that is all days with more precipitation than the first quartile (= above 0.15 mm d^−1^), as well as days after such precipitation events. Water content fluctuations in the soil after such rewetting precipitation events were likely affected by soil water redistribution through percolation rather than root water uptake and were therefore not suitable to estimate root water uptake dynamics. We fitted gamma distributions (a suitable distribution to model time durations) to the timing of daily maximum sap flow and root water uptake except for sap flow measured at beech because of its bimodal daily dynamics, causing the maximum to occur either in the morning or in the afternoon. A nonparametric univariate kernel density estimator was used to fit the bimodal distribution of beech sap flow.

We define capacitance as the derivative of plant water storage by plant water potential *C* = d*V*_cap_/dѰ_cap_. Capacitance in the roots was measured on excavated and cut root segments of beech and spruce trees in the laboratory. We cut root segments of *c*. 10 cm length and 3–4 cm diameter, saturated them in water for 24 h. We then installed psychrometers (PSY1, ICT International, Armidale, NSW, Australia) for continuous water potential monitoring, placed the root segments on scales and recorded the weight change over time to monitor the water content of the root segments. We report capacitance of two root segments per species.

We estimated main physical properties of the soils at our site by sampling soil close to beech and spruce trees at different depths. Saturated hydraulic conductivity was measured with a permeameter (Royal Eijkelkamp, Giesbeek, the Netherlands), soil water retention curves and unsaturated hydraulic conductivity were measured with the HYPROP 2 device (Meter Group GmbH) at 10, 20 and 40 cm depths (more detailed procedures and results from the analyses are reported in Methods S3). We used these measurements to parameterize the soil component of the soil–plant hydraulic model.

### The soil–plant hydraulic model

**Fig. 2:**
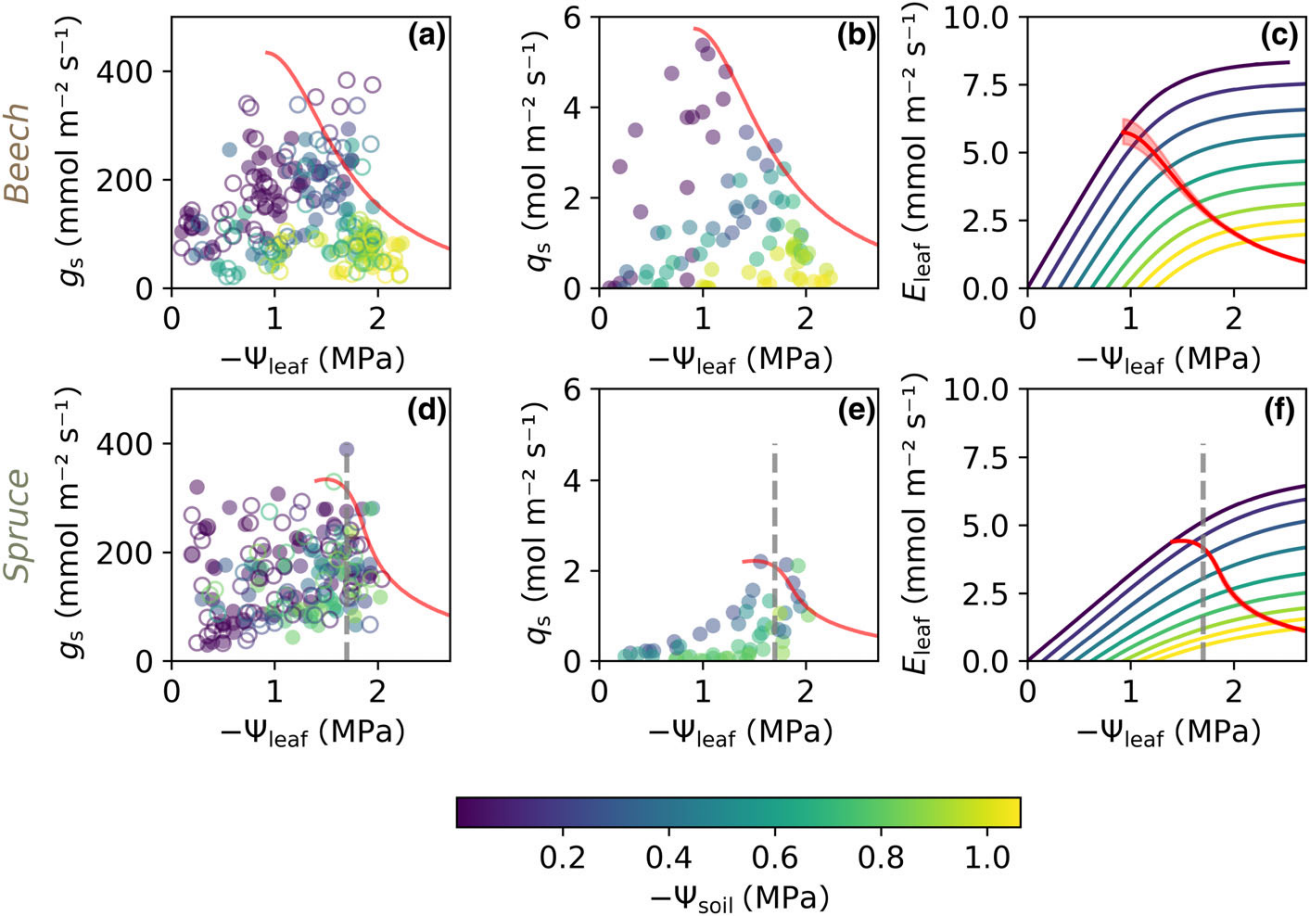
Observations (markers) and simulations (lines) of soil–plant hydraulic dynamics and of beech (a–c) and spruce (d–f). Measurements of leaf water potential Ѱ_leaf_ (*x*‐axis) along with stomatal conductance *g*_s_ (a, d) indicate different strategies of stomatal closure of beech and spruce trees causing comparable dynamics in sap flow *q*_s_ (b, e). The different trees where stomatal conductance and leaf water potential were measured are indicated by filled and empty circles. Stomatal conductance measurements were used to calibrate a soil–plant hydraulic model that relates soil and leaf water potentials to transpiration (*E*_leaf_) without accounting for capacitance (c, f). The colored lines in (c, f) indicate how leaf water potential responds to linearly increasing transpiration for an initial soil water potential. The red curves occur at the maximum *E*_leaf_/Ѱ_leaf_ along increasing transpiration and represent the onset of stomatal closure well aligned with measurements at beech trees (a, b). Shading indicates the parameterization obtained from individual trees, which were averaged to obtain a species‐specific parameterization. The dashed gray line represents an alternative stomatal regulation strategy, where onset of stomatal closure occurs at a fixed Ѱ_leaf_ threshold (here −1.7 MPa), well aligned with measurements at spruce trees (d, e). Mean average errors between observed and simulated leaf water potentials and transpiration *E*_leaf_ are listed in Supporting Information Table S2, and a comparison between measured and simulated dynamics of leaf water potential are shown in Fig. S4. The measured data are available in Supporting Information.

**Fig. 3:**
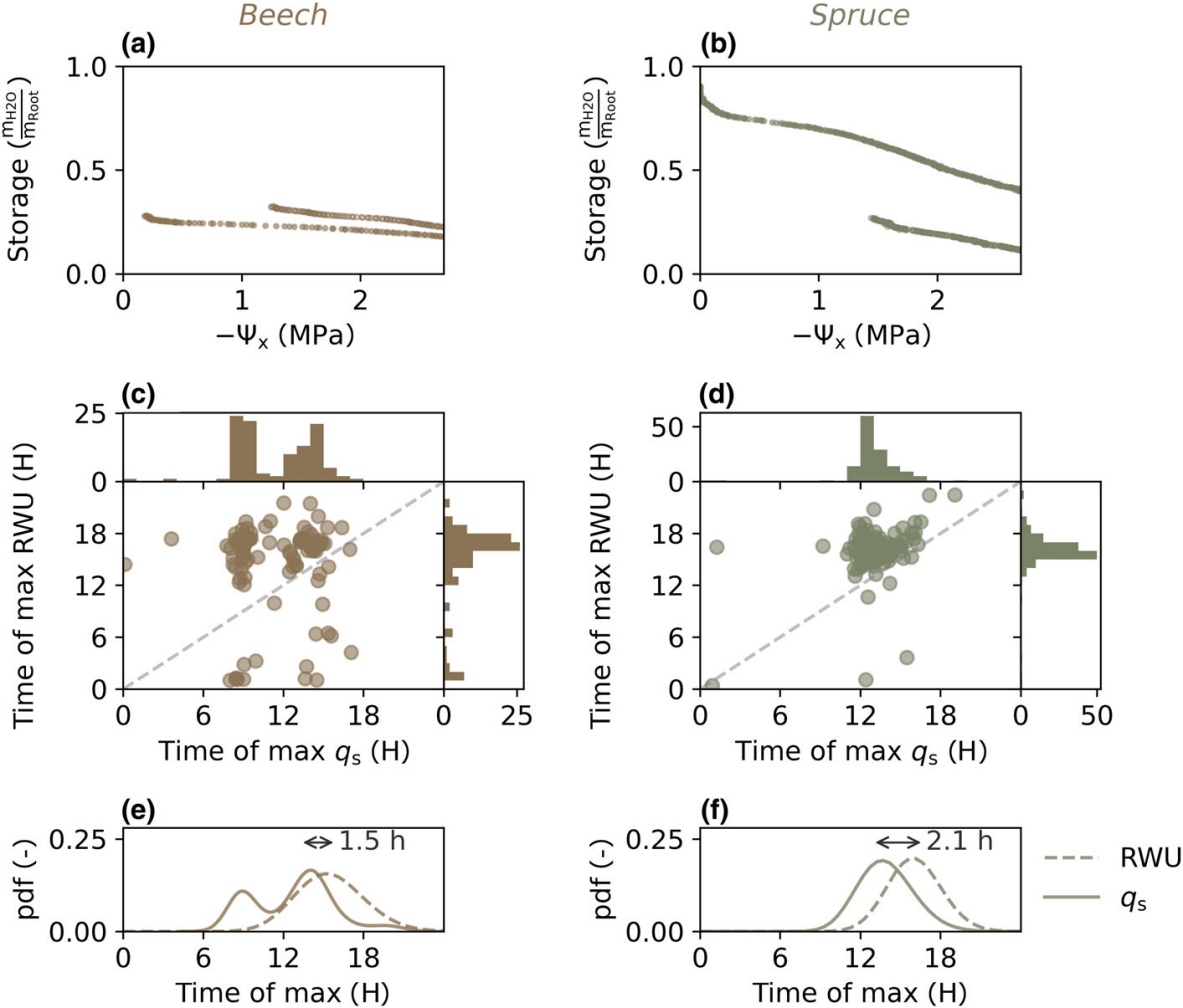
Hydraulic capacitance (a, b) measured on root segments by relating water relative storage (*y*‐axis) to xylem water potential Ѱ_*x*_ (*x*‐axis). Relative storage equals the water mass (m_H2O_ (g)) divided by the dry weight of the root segment (m_Root_ (g)). Timings (c, d) of the maxima in sap flow *q*_s_ (*x*‐axis) and root water uptake (RWU) estimated from soil water content at 40 cm depth (*y*‐axis) for beech (c – left panel) and spruce (d – right panel), along with their respective histograms. Fitted probability density distributions (pdf – e, f) indicate the average time lags between maxima of sap flow and root water uptake, resulting from capacitance. (c, d) show data of days when both sap flow and root water uptake were computed, while probability density functions (gamma distribution for root water uptake and spruce sap flow and kernel density estimation for beech sap flow) in (e, f) were estimated from all data obtained during the growing seasons (April–September) of 2022 through 2024. Similar results for individual sensors at 10, 20 and 80 cm are shown in Supporting Information Figs S7–S9. Spruce showed larger capacitance on root segments (a, b) and longer time lags between daily peaks in sap flow and root water uptake (c–f).

The original soil–plant hydraulic model (Carminati & Javaux, 2020) assumes steady‐state water flow from the bulk soil via the roots (implemented as a single long root) to the leaves (implemented as a single big leaf) driven by the gradient in water potential along these compartments, without considering plant water storage and capacitance. In reality, plants can source water (1) stored within their xylem tissues (apoplastic water) to supply transpiration and (2) stored within cells (symplastic water) to support hydraulic functioning even after water transport from the roots has been disrupted (Mantova *et al*., 2022). We therefore extended the original model with plant water storage that can be sourced for transpiration. We assumed a bulk water storage across the plant that supports the transpiration stream concomitant to root water uptake. This implies that the water fluxes from plant water storage and the water fluxes from the soil via root water uptake are both driven by xylem water potential (see Fig. 1 – schematics of the extended model). In the model, capacitance is assumed to be constant along varying water potential (i.e. we assume a linear relationship between plant water storage and water potential), because we observed vastly linear relationships between water storage and water potential within the range at which plants typically operate during daytime transpiration (−1 to −2 MPa, see Figs 2, 3a,b). A linear relationship between storage and potential allows us to vary capacitance by either varying the maximum stored water volume (storage capacity) or by varying the minimum water potential at which the stored water is drained to a minimum. We analyzed the sensitivity of soil–plant hydraulic simulations to capacitance by varying plant water storage capacity and fixing the minimum water potential to −4.5 MPa. This water potential corresponds to double the minimum leaf water potential that was measured in the field (−2.25 MPa, see Fig. 2).

To simulate soil–plant hydraulic dynamics, we imposed evaporative demand (as a boundary condition reflecting potential transpiration), initial soil–water availability (conditions at zero transpiration) and assumed that the plant (including the plant water storage compartment) is in equilibrium with the soil at the onset of transpiration. At each time step, the model iteratively solves for the xylem water potential that partitions the imposed transpiration into root water uptake from the soil (*Q*_soil_) and the water flux from plant water storage (*Q*_cap_). The model first calculates *Q*_cap_ from the gradient between the plant water storage potential and xylem potential (taken either from the previous iteration or from the previous time step or iteration) with Eqn 1 (see Fig. 1). This flux is then subtracted from the imposed transpiration to obtain *Q*_soil_. The obtained root water uptake flux implies a certain water potential at the root–soil interface (according to Eqn 3), that is subsequently used to calculate the xylem water potential for the next iteration (according to Eqn 2). To avoid numerical instabilities, we averaged this new xylem water potential with the one from the previous iteration and stopped the iteration once subsequent xylem water potentials were less than 5 × 10^−5^ MPa apart from each other. At each timestep, the calculated flux volumes *Q*_cap_ and *Q*_soil_ were subtracted from the soil water and the plant water storage volumes (according to Eqn 4a,b), respectively, and the resulting water potentials were estimated from the soil water retention curve (Fig. S2) and the imposed capacitance (*C* = d*V*_cap_/dѰ_cap_). Methods S4 contain a detailed model description.

In this study, we show the model results along with the observation data to (1) test whether model simulations capture soil–plant hydraulics of beech and spruce trees, (2) define stomatal regulation strategies based on observed and simulated soil–plant hydraulics and (3) assess how different plant water capacitances and contrasting stomatal regulation strategies affect soil–plant hydraulics under varying soil water conditions.

### Onset of stomatal closure

**Fig. 4:**
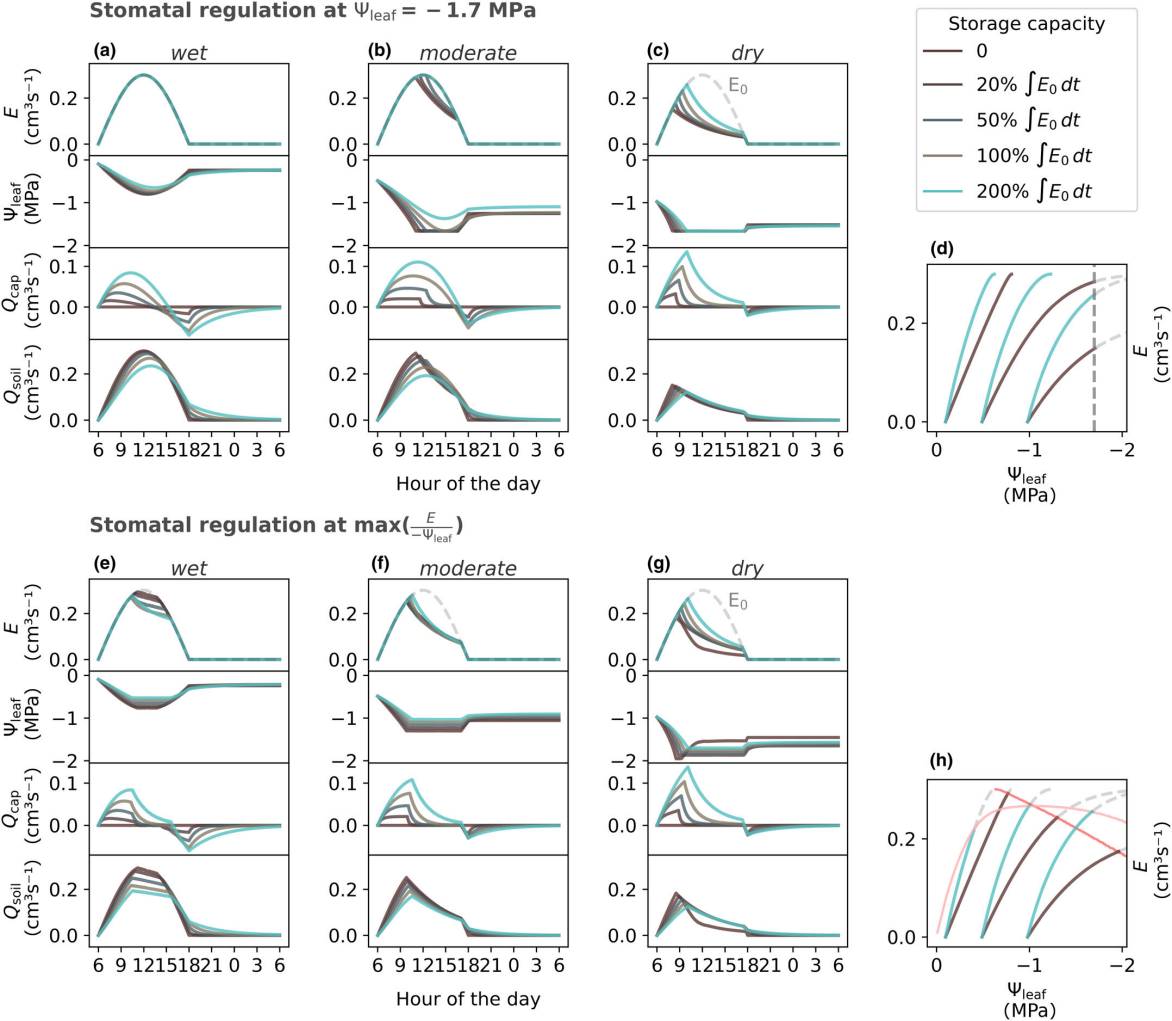
Soil–plant hydraulic simulations of transpiration *E*, leaf water potential Ѱ_leaf_, water fluxes sourced from plant storage *Q*_cap_ and root water uptake *Q*_soil_, for a generic average plant calibration with different amounts of plant water storage capacities and different initial soil water contents. When fluxes are positive, water for transpiration is drawn from the respective storages (plant water storage *Q*_cap_ and soil water storage *Q*_soil_). (d, h) show the relationship between transpiration and leaf water potential, which for clarity are only shown for storage capacities equal to 0 and 200% the evaporative demand (*E*_0_) integrated over a day and only while transpiration increases. For the simulations in (a–d) (top row), the onset of stomatal closure occurs at a fixed leaf water potential of −1.7 MPa. For the simulations in (e–h) (bottom row), the onset of stomatal closure occurs when *E*/−Ѱ_leaf_ starts to decrease with increasing *E* (= flexible strategy). The red curves in (h) (lighter shade indicates higher capacitance) show the onset of stomatal regulation for a flexible strategy, occurring at maximum *E*/−Ѱ_leaf_, which is linked to the nonlinearity between *E* and Ѱ_leaf_.

Solving the soil–plant hydraulic model reveals the water potential along the different compartments of the soil–plant hydraulic system (including the plant water storage compartment). The model also provides the partitioning between root water uptake and plant water storage, without accounting for any direct regulation of transpiration by stomata. When plotting transpiration against the calculated leaf water potential for varying soil water potential (Figs 2, S3), the resulting two‐dimensional space can be divided into two regimes: (1) a linear regime, where transpiration is linearly dependent on leaf water potential and (2) a nonlinear regime, where increasing transpiration cause nonlinear responses in leaf water potential (see Fig. 2). Here, we considered that the onset of stomatal closure occurs when leaf water potentials (Ѱ_leaf_) reach certain limits (Ѱ_leaf,lim_) and that stomatal regulation keeps Ѱ_leaf_ above Ѱ_leaf,lim_, resulting in constant Ѱ_leaf_ once stomata regulate transpiration (see Fig. 4). Indeed, measurements of leaf water potential during dry conditions showed relatively constant values during periods of stomatal regulation of transpiration (Fig. S4b,d) We tested two possibilities to determine Ѱ_leaf,lim_, corresponding to two contrasting stomatal regulation strategies observed for beech and spruce trees. The first strategy sets the onset of stomatal closure at a constant leaf water potential threshold, independently of soil water potential, therefore corresponding to a relatively isohydric stomatal regulation strategy (as observed for spruce). The second strategy sets the onset of stomatal closure to maximize transpiration over leaf water potential, that is plants control transpiration by keeping stomata open in the linear regime of the relationship between transpiration and leaf water potential, and close stomata when limited water supply causes nonlinearity between transpiration and leaf water potential (Sperry & Love, 2015; Carminati & Javaux, 2020). For a given soil water potential and evaporative demand, the limit leaf water potential Ѱ_leaf,lim_ occurs when the ratio of E to −Ѱ_leaf_ is maximal. *E* at Ѱ_leaf,lim_ approximates the change from a linear to a nonlinear regime (red curves in Figs 2c,f, 4h). Numerically, we maximize *E*/(−Ѱ_leaf_ + ԑ) and use a threshold of ԑ = 0.2 MPa, to allow transpiration under wet soil conditions, otherwise the maximum *E*/−Ѱ_leaf_ would be reached at relatively low transpiration and an excessively early onset of stomatal closure. The onset of stomatal closure occurs at leaf water potential Ѱ_leaf,lim_ that decreases with dropping soil water potential, which corresponds to a more anisohydric stomatal regulation strategy (as observed for beech).

### Model parameterization, calibration and validation

A complete list of model parameters and used values is available in Table S1. We set the Brooks‐Corey exponent for the xylem and the root radius to realistic values used in previous studies (Abdalla *et al*., 2022; Wankmüller & Carminati, 2022). Model calibration involved the maximum soil–plant conductance (*K*_*x*0_), the length of roots for water uptake (L), and the xylem water potential at which the xylem hydraulic conductance starts to decrease (*h*_*x*0_). We used measurements of leaf transpiration and leaf water potential from individual trees at our site to find tree‐specific calibrations and averaged them across trees of the same species to obtain species‐specific parameters. For the calibration of the soil–plant hydraulic model we neglected soil drying (i.e. soil water potential remains constant along increasing transpiration) and capacitance, because these would require calibration of further parameters (accessible soil volume, plant water storage capacity and capacitance), that cannot be achieved meaningfully with the available data. Root‐accessible soil volume was set to 1/3 m^3^. With a saturated soil water content of 53.3%, a water content at the wilting point (−1.5 MPa) of *c*. 21% and a residual water content of 3% (see soil retention curve – Fig. S2), this soil volume holds the equivalent water volume of 12 or 20 d of unrestricted sinusoidal evaporative demand with a maximum rate of 0.3 cm^3^ s^−1^, depending on whether the wilting point or the residual water content is considered. The radius of the soil surrounding the root was then calculated from the accessible soil volume, the root length and the root radius of 0.5 mm. It is important to note that the soil–plant hydraulic model relates water flux to water potential at the leaf and does not directly calculate stomatal conductance. Therefore, we converted our stomatal conductance measurements to leaf transpiration *E*_leaf_ in order to compare model simulations to measurements during the calibration procedure. We estimated leaf‐level transpiration *E*_leaf_ by multiplying measured stomatal conductance with VPD at the leaf divided by atmospheric pressure at the time of the measurement (further explained in Methods S1).

For each tree, we estimated the parameter *K*_*x*0_ by dividing the maximum measured leaf transpiration *E*_leaf_ by the corresponding leaf water potential Ѱ_leaf_, thereby assuming that during this measurement soil water potential was negligibly high (Fig. S3) and stomata allowed transpiration flux under no loss of hydraulic conductivity in the xylem. The parameters *L* and *h*_*x*0_ were estimated by fitting a curve indicating the onset of stomatal closure to a subset of the observations, for which we assumed that the data are close to maximal stomatal conductance for given soil and leaf water potentials (see red curve in Fig. 2c,f and the previous ‘Onset of stomatal closure’ in the Material and Methods section). To select the data, we binned the *E*_leaf_ measurements in 8 bins of equal width and selected for each bin the *E*_leaf_ measurement with the lowest (most negative) leaf water potential Ѱ_leaf_ but neglected leaf water potentials that were less negative than −1.5 MPa. For the calibration, we simulated the soil–plant hydraulics for the evaporative demand linearly increasing from 0 to 0.9 cm^3^ s^−1^ and soil water potential ranging from 0 to −2 MPa. The values *L* and *h*_*x*0_ were calibrated by iterating through different combinations of *L* and *h*_*x*0_, choosing the simulation with the lowest sum of squared normalized errors between the onset of stomatal closure (calculated by the maximization strategy described further above) and the selected data points, in both directions, leaf transpiration *E*_leaf_ – *y*‐axis, and leaf water potential Ѱ_leaf_ – *x*‐axis. The errors were normalized by the measured maximum *E*_leaf_ and minimum Ѱ_leaf_, respectively. The estimated onset of stomatal closure is displayed along stomatal conductance measurements and sap flow data (Fig. 2a,b,d,e). For the stomatal conductance data, the simulated onset of stomatal closure was multiplied by the ratio between average atmospheric pressure and average VPD during all stomatal conductance measurements (this is the inverse procedure compared to obtaining leaf level transpiration described further above). For sap flow data, the simulated *E*_leaf_ was multiplied by an empirical factor representing the fraction between leaf area and sapwood area. This factor was not measured (since we did not directly measure leaf area nor sapwood area) but was obtained by matching the maximum in simulated and measured sap flow (we used factors equal to 1000 and 500 for beech and spruce, respectively, see Fig. 2b,e). We additionally validated the model's ability to predict leaf water potential by comparing simulated and measured subdaily leaf water potential dynamics. We selected 2 d with different soil water conditions but very similar atmospheric conditions (Fig. S5), for which sap flow velocity and subdaily measurements of leaf water potential were available for beech and spruce trees (9 and 16 August 2022). We used sap flow as a proxy for the evaporative demand on these days (rescaled such that maximum evaporative demand equals 0.3 cm^3^ s^−1^ in order to match the maximum transpiration rate of the sinusoidal evaporative demand) and predawn leaf water potentials as initial conditions of the soil water potential. This analysis is shown in Fig. S4.

**Fig. 5:**
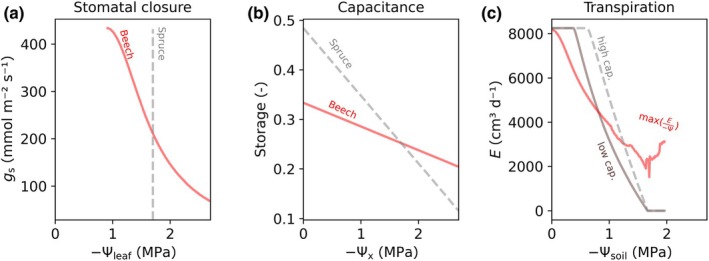
Comparison of (a) stomatal regulation strategy (variable or fixed leaf water potential Ѱ_leaf_ at the onset of stomatal closure g_s_) and (b) capacitance (water storage emptying with dropping xylem water potential Ѱ_*x*_) for beech (red) and spruce (gray). (c) Simulated daily transpiration fluxes *E* for drying initial soil water conditions determined by the soil water potential Ѱ_soil_ and parameterizations corresponding to a generic plant with a fixed leaf water potential at the onset of stomatal closure and small capacitance (brown – ‘strict’ stomata and low capacitance), large capacitance (gray dashed – ‘strict’ stomata and large capacitance) and variable leaf water potential at the onset of stomatal closure and small capacitance (red – ‘flexible’ stomatal regulation strategy and small capacitance).

We performed a sensitivity analysis between transpiration, plant water storage capacity and capacitance for the two different stomatal regulation strategies. For these simulations, we used an average tree calibration (average of calibrated model parameters for beech and spruce) and a evaporative demand with sinusoidal dynamics. We varied plant water storage capacity by multiplying the total daily transpiration flux with different factors (0, 20, 50, 100, 200%). Thus, the maximum ratio between whole‐plant water storage to soil water storage in our simulations was roughly 1/10, which is well aligned with recent global estimates of aboveground plant water and soil water storages (global average ratio of 1/34 estimated by Felton *et al*., 2025). To obtain capacitance we assumed a linear pressure–volume relationship and assumed plant water storage to be completely emptied at a water potential of −4.5 MPa (see ‘The soil–plant hydraulic model’ in the Materials and Methods section). We ran the model for different soil water potentials (initial conditions) and for two different stomatal regulation strategies, reflecting the observed isohydric and anisohydric behavior of spruce and beech, respectively, and analyzed the effects of varying capacitance on subdaily and daily transpiration dynamics (Figs 4, 5). It is not our objective to use the model to accurately predict soil–plant hydraulics of beech and spruce trees, but rather to explore the interaction between stomatal regulation strategy and hydraulic capacitance.

## Results

### Observed transpiration dynamics and soil–plant hydraulic simulations

Measurements of stomatal conductance and leaf water potential revealed an onset of stomatal closure for beech trees that was strongly linked to soil water availability. Leaf water potential as well as stomatal conductance decreased with dropping soil water potential as an effect of soil drying (Fig. 2a). Although spruce and beech trees are growing close to each other at our site, spruce trees experienced relatively wetter soil due to lower root water uptake and lower overall transpiration (see also Martinetti *et al*., 2025). Therefore, we measured stomatal conductance under less dry soil conditions for spruce compared to beech. Beech showed sap flow and stomatal conductance measurements dropping at low soil water potential (Fig. 2a,b), while spruce stomatal conductance and sap flow dynamics showed lower covariation with soil water potential (Fig. 2e,f). Overall, the simulated soil–plant hydraulics well reproduced the higher transpiration for beech compared to spruce. The simulated onset of stomatal closure that was obtained from the calibrated soil–plant hydraulic models by maximizing *E*/−Ѱ_leaf_ (Fig. 2c,f) corresponds well to the observed stomatal conductance and sap flow dynamics for beech. Spruce, however, showed a more stringent (more isohydric) stomatal regulation strategy and kept leaf water potentials close to −1.7 MPa, potentially avoiding low soil water potential as a result of this strategy (gray dashed line in Fig. 2d,e). The parameter values for the soil–plant hydraulic simulations were obtained by calibrating to observations of leaf transpiration *E*_leaf_ and leaf water potential Ѱ_leaf_ and are reported in Table 1. Readers might notice the large value for the root length *L*. This large value is a consequence of the simplified geometry of the single root approach, along with the fact that capacitance is not considered for model calibration. This results in exaggerated root length compensating for the lack of capacitance. The validation of the model simulations captured the general dynamics in leaf water potential (Fig. S4), but with relatively high uncertainty. We used measured sap flow dynamics to infer the evaporative demand, however, sap flow measurements at the stem mask potential contributions from capacitance to transpiration above the location of the sensor (i.e. breast height), thereby likely underestimating transpiration and overestimating simulated leaf water potentials. Similarly, time lags between simulated and observed daily minimum leaf water potentials (particularly noticeable in Fig. S4c) likely result from sap flow measurements at the stem not corresponding to overall transpiration dynamics for trees with large capacitance.

**Table 1:** Average calibrated parameters for the species‐specific soil–plant hydraulic simulations.

Parameter	Value	Unit
Max. soil–plant hydraulic conductance *K*_*x*0_	0.71 (Beech) 0.35 (Spruce)	cm^3^ (MPa s)^−1^
Root length *L*	27 375 (Beech) 10 125 (Spruce)	m
Xylem water potential at onset of loss of conductivity *h*_*x*0_	−1.7 (Beech) −2.8 (Spruce)	MPa

A complete list of the parameters including tree‐level calibration values are listed in Supporting Information Table S1. Validation of daily dynamics of leaf water potentials are shown in Fig. S4.

### Capacitance and its effects on soil–plant hydraulics

Measurements (Fig. 3a,b; Table S3) revealed relatively large capacitance for spruce (0.136 kg MPa^−1^ kg^−1^) compared to beech (0.048 kg MPa^−1^ kg^−1^), as indicated by the steeper slope between water potential and plant water storage measured on two root segments of beech and spruce (see also Martinetti *et al*., 2025). On average, capacitance (the slope of the regression lines, see Fig. 5b) was 2.85 times larger for spruce than for beech. For spruce, sap flow typically peaked around midday and the timing of the maximum tended to occur earlier when the soil was relatively dry (Figs 3c,f, S6). Maximum root water uptake of spruce occurred on average *c*. 2 h later than maximum sap flow (Fig. 3f). For beech, the daily sap flow cycle typically showed two peaks (Fig. 3c,e). The timing of maximum sap flow was highly dependent on soil water content: when soil was relatively dry, daily maxima occurred in the morning, that is roughly between 08:00 h and 10:00 h; when soil was relatively wet, daily maxima occurred during the afternoon, that is 13:00 h–15:00 h (Fig. S6). However, beech root water uptake showed only one peak, which typically occurred between 16:00 h and 18:00 h (Fig. 3c). The average time lag between maxima of beech sap flow and beech root water uptake was 1.5 h and therefore smaller (albeit more variable) compared to spruce. Without capacitance, the two distributions of maximum sap flow and root water uptake should broadly overlap. Accordingly, the time lag between the maxima of sap flow and root water uptake becomes larger with greater capacitance (see also ‘Effects of varying levels of plant water storage and capacitance on transpiration’ section on model temporal dynamics) as water is sourced from internal water storage instead of from root water uptake, causing root water uptake to occur later compared to sap flow. The observed time lags between maxima of sap flow and root water uptake also suggest larger capacitance for spruce compared to beech.

### Effects of varying levels of plant water storage and capacitance on transpiration

We used sinusoidal transpiration dynamics to run the model (evaporative demand) for varying levels of capacitance and varying soil water conditions and used the stomatal regulation strategies to limit leaf water potential Ѱ_leaf_ and actual transpiration *E*. When leaf water potential was above (less negative than) the threshold obtained from the stomatal regulation strategy, the tree could satisfy the evaporative demand and stomata were not regulating transpiration. Contrary to this, leaf water potential falling below the thresholds obtained from the stomatal regulation strategy caused stomatal closure and transpiration reduction until the leaf water potential threshold was satisfied (see also ‘Onset of stomatal closure’ in the Materials and Methods section). The model additionally partitioned transpiration *E* into internal plant water storage *Q*_cap_ and root water uptake *Q*_soil_ (Fig. 4). Generally, for both stomatal regulation strategies, increasing capacitance led to the following observations:

1. The larger the capacitance, the higher (less negative) the required leaf water potential to sustain a certain transpiration.
2. The larger the capacitance, the larger the lag time between transpiration *E* and root water uptake *Q*_soil_, because the fraction of transpired water sourced from plant water storage increased with increasing capacitance, and root water uptake shifted to a later time.
3. On a daily timescale, the soil water budget was largely unaffected by capacitance. However, during times of high transpiration, less soil water was being sourced as capacitance increased (due to point 2).
4. Larger capacitance resulted in higher water fluxes from the soil to the plant during the night, as a consequence of higher root water uptake required to refill internal plant water storage that was emptied during the day through transpiration. When soil was dryer, nocturnal water fluxes were smaller, as the water potential gradient between the roots and soil decreased.

Besides these similar responses, the two stomatal regulation strategies had different effects on soil–plant hydraulic dynamics. For plants closing stomata at a fixed leaf water potential, the (fixed) leaf water potential threshold was not reached for any of the capacitance scenarios under wet soil conditions, causing no reduction in transpiration (Fig. 4a). However, the sensitivity of transpiration to capacitance increased with soil drying, and increasing capacitance caused a slower drop of leaf water potential, due to the increased fraction of internally stored water used for transpiration, which could sustain the evaporative demand for a longer time (Fig. 4b,c). For plants using a flexible strategy, larger capacitance caused lower transpiration due to earlier stomatal closure in wet soil (Fig. 4e). Increasing capacitance led to a relatively higher degree of nonlinearity in the relationship between transpiration *E* and leaf water potential Ѱ_leaf_ because of the slow decline in leaf water potential under rising *E* (at the beginning of the day) supported by the higher depletion of internal water storage (Fig. 4h). Progressive emptying of plant water storage caused its water potential to further decline, thereby limiting the contribution of plant water storage to transpiration. As evaporative demand further increased, leaf water potential started to decline more rapidly, leading to earlier occurrence of maximum *E*/−Ѱ_leaf_ and subsequent regulation of transpiration *E*. This process was dominant when the soil was wet and the plant internal water storage full, allowing plants to source large amounts of water from internal water storage. Under moderate soil water conditions (soil water potential of *c*. −0.5 MPa), the flexible stomatal regulation strategy showed low sensitivity to changes in capacitance (Fig. 4f). Under dry soil conditions, the simulations were similar for both stomatal regulation strategies, suggesting that transpiration was limited independently of stomatal regulation strategy (Fig. 4c,g).

Simulated daily transpiration volumes varied based on stomatal regulation strategy and degree of capacitance corresponding to beech and spruce trees (Fig. 5). Generally, the flexible stomatal regulation strategy, as observed on beech trees, caused lower daily transpiration under wet conditions (here for soil water potential above −0.8 MPa) because of stomatal closure occurring relatively early in the day (see Fig. 4e). However, under drying soil, the flexible stomatal regulation strategy became beneficial, that is yielded higher transpiration than when the onset of stomatal closure occurred at a fixed leaf water potential because leaf water potential could drop further and thereby the plant could sustain the evaporative demand for longer times throughout the day. Increasing capacitance with a strict stomatal regulation strategy, as observed on spruce trees, allowed higher transpiration when soil water potential decreased (see Fig. 5c). With larger capacitance, leaf water potentials dropped less rapidly and reached the onset of stomatal closure later during the day, independently from soil water availability. The resulting relationship between soil water potential and transpiration E shifted to the right and became (more) linear with larger capacitance, because capacitance buffered strong reductions in transpiration that would have occurred under limiting soil water availability. Contrary, larger capacitance for the flexible stomatal regulation strategy would have increased the nonlinearity between soil water potential and daily transpiration due to the reduced transpiration under wet conditions, but increased transpiration under dry conditions (for the sake of clarity not shown in Fig. 5, but observed in Fig. 4e–g).

This analysis reveals interactions as well as similarities between capacitance and flexible stomatal regulation. Both large capacitance and flexible stomatal regulation enable plants to sustain transpiration during soil drying. However, the combination of the two traits (i.e. if a plant has both large capacitance and flexible stomatal regulation) might reduce the effect of each isolated trait (in wet soil).

## Discussion

There have been several efforts to include hydraulic capacitance in numerical soil–plant hydraulic models (e.g. Tyree, 1988; Kumagai, 2001; Manoli *et al*., 2017; Huang *et al*., 2017; Xie *et al*., 2023; Alléon *et al*., 2025). All these models (including ours) describe water flow along the soil–plant‐atmosphere continuum with the cohesion–tension theory combined with an Ohm's law analogy of water flow along a sequence of resistors and capacitors. Our model uses a single big leaf, single long root and single capacitor approach, and therefore does not describe heterogeneities of a tree's canopy (Tyree & Ewers, 1991; Früh & Kurth, 1999; Bittner *et al*., 2012; Silva *et al*., 2022) or root system (Couvreur *et al*., 2012; Xu *et al*., 2016; Huang *et al*., 2017), nor the difference in capacitance between symplast and apoplast (Ruffault *et al*., 2022). We quantified capacitance with a linear pressure–volume relationship, because in the range of the observed daily minimum leaf water potentials (−0.8 to –2.2 MPa) the capacitance measurements showed a linear relationship (see Fig. 3e,f). However, there are likely differences in capacitance and xylem vulnerability across tree compartments (Mcculloh *et al*., 2014; Domec & Gartner 2001), which can vary along different ranges of water potential (Salomón *et al*., 2017; Hernando *et al*., 2025; Zhang *et al*., 2025). Our model also lacks explicit modelling of physiological processes that interact with transpiration, for example hormonal ABA signaling (McAdam & Brodribb, 2016). However, our model is easily scalable across tree size by factoring imposed transpiration *E* and the parameters describing root length (*L*), whole‐plant conductance (*K*_*x*0_), plant water storage (*V*_cap_) and accessible soil volume (*V*_s_).

Transpiration is driven by highly correlated variables such as atmospheric VPD and solar radiation, which in turn are heterogeneous across tree canopies and affect stomatal closure at the single leaf (Ball *et al*., 1987; Yi *et al*., 2020), while our approach considers stomatal closure as a purely hydraulic phenomena without accounting for energy limited stomatal closure. We implemented two contrasting stomatal regulation strategies based on measurements performed on beech and spruce trees (‘strict’/isohydric and ‘flexible’/anisohydric stomatal regulation at constant and variable leaf water potential thresholds, respectively). These models are not meant to be mechanistic representations of the two specific tree species, but to effectively simplify soil–plant hydraulic dynamics in order to improve our understanding of the interaction between stomatal regulation strategy and hydraulic capacitance. Shifts between relatively more isohydric and anisohydric water use strategies, caused by fluctuations in environmental variables, have been observed (Feng *et al*., 2019; Novick *et al*., 2019). Comparing beech and spruce observations indicates that spruce limits transpiration with a relatively strict stomatal regulation strategy. However, spruce might have adapted a flexible strategy under wet conditions and switched to a strict stomatal conductance strategy when soil water potential decreased, as also suggested by sap flow measurements in Fig. 2(e). Hypothetically, a shift from a flexible to a strict stomatal regulation as soil dries would explain how spruce, despite having higher capacitance and water storage, regulated transpiration more rigorously even under wet conditions and thereby conserved soil water for longer.

Several studies concluded that the effect of capacitance on soil–plant hydraulic and ecosystem functioning in temperate climates is minor at larger timescales, but important to capture dynamics at the sub‐daily timescale, particularly under limiting soil water conditions (Carlson & Lynn, 1991; Liu *et al*., 2021; Paschalis *et al*., 2023). Plants have been shown to switch to internal plant water storage to source water for transpiration during atmospheric drought (Matheny *et al*., 2015; Bryant *et al*., 2021; Guo *et al*., 2024). As observed in our simulations, at shorter timescales, plant water storage buffers leaf water potential because water sourced from internal plant water storage has smaller resistance than water sourced from the soil. This allows a slower decline of leaf water potential and respective maintenance of stomatal aperture and transpiration. At longer timescales, however, larger capacitance would generally not substitute for a lack of available soil water in temperate climates. While, with larger capacitance, the rate of root water uptake is lower during the day when transpiration occurs, the rate of root water uptake is higher during the night when depleted plant water storage gets refilled, broadly compensating the soil water savings during the day from larger capacitance (Tyree & Ewers, 1991). Studies have highlighted the importance for trees to refill water storage overnight, and that the failure to refill water storage affects daytime stomatal regulation (Scholz *et al*., 2007; Peters *et al*., 2023, 2025). Peters *et al*. (2025) detected a uniform threshold in predawn leaf water potential at −1.2 MPa causing strong daytime regulation of transpiration across species, whereas midday leaf water potential showed higher variability. Our model shows that under dry conditions (predawn water potential of −1 MPa, see Fig. 4c,g), simulated transpiration sensitivity to stomatal regulation strategy decreased, with strongly limited transpiration independent of stomatal regulation strategy and degree of capacitance. While larger capacitance and plant water storage enabled higher transpiration by buffering leaf water potentials for both stomatal regulation strategies, transpiration did not vary substantially between stomatal regulation strategies and did not meet the evaporative demand. This supports the hypothesis of a uniform threshold in predawn leaf water potential coinciding with stronger stomatal closure during the day.

Nevertheless, species with different stomatal regulation strategies and different capacitances might increase ecosystem productivity because of their contrasting water usage and potentially reduced intraspecific competition for water. Optimization principles have shown that solely the presence or absence of competitive water sinks can already alter stomatal regulation and ecosystem transpiration significantly (Mrad *et al*., 2019). Our field site is a mixed temperate forest with beech trees reducing soil matric potential more rapidly than spruce trees due to the relatively flexible stomatal regulation. It is plausible that a pure beech forest would generally encounter droughts more frequently, given the tendency to use more water with progressing soil drying, while a pure spruce forest would encounter droughts less frequently but would also have slower growth (due to stronger regulation of transpiration) despite favorable conditions.

Our analysis suggests a coordination between capacitance and stomatal regulation strategy, as also found in previous studies (Hartzell *et al*., 2017; Matheny *et al*., 2017; McCulloh *et al*., 2019). From a theoretical perspective on soil–plant hydraulics and drought resistance, it is not always convenient to employ high capacitance, due to the generally increased water consumption and slower nocturnal recovery, in particular for plants with a flexible stomatal regulation strategy. Indeed, sapwood capacitance has been shown to be larger at wetter sites (Richards *et al*., 2014). Optimality principles have also indicated how long‐term development of functional hydraulic traits enabling higher stomatal conductance under drought, such as xylem resilience, might be constrained by nonstomatal limitations of photosynthesis induced by low leaf water potential (Matthews *et al*., 2024). On another level, high capacitance requires more plant tissue to have low density (including sapwood), which in turn would generally make plants less resistant to embolism (Meinzer *et al*., 2009; Fu & Meinzer, 2019; McCulloh *et al*., 2019), and more susceptible to tissue shrinkage during times of water loss. This could lead to higher shrinkage rates of roots when water is drawn from their internal storage, causing earlier decoupling of the soil–root interface and thereby abrupt decline in the soil–root hydraulic conductance (Nobel & Cui, 1992; Carminati *et al*., 2009). Coordination between stomatal regulation strategies and capacitance is therefore an integral part of the coordination of stomatal and xylem functioning (Sperry, 2004) and might be essential in controlling transpiration across different species and environments (Gleason *et al*., 2014).

We have shown the soil–plant hydraulic interplay between species‐specific plant‐hydraulic traits, stomatal regulation strategy and capacitance by extending a simple soil–plant hydraulic model. We tested two different strategies of leaf water potential regulation by stomata and assessed the soil–plant hydraulic sensitivity to capacitance by varying the size of internal plant water storage capacity. Overall, large capacitance allows plants to source higher fractions of water from internal plant water storage. Plants with a stomatal regulation strategy that prevents leaf water potential from dropping below a fixed threshold benefit from large capacitance due to the buffering effect on leaf water potential. On the contrary, a stomatal regulation strategy that maximizes transpiration with decreasing leaf water potential responds to large capacitance with an earlier stomatal downregulation under wet conditions. Our analysis highlights a trade‐off between hydraulic traits of stomatal regulation and capacitance in temperate ecosystems.

## Competing interests

None declared.

## Author contributions

SM, AC, PM and MGF designed the research and interpreted the results. SM collected the data, implemented plant water storage into the model and ran the simulations. SM produced the figures and wrote the initial draft of the manuscript. All authors revised and approved the manuscript.

## Disclaimer

The New Phytologist Foundation remains neutral with regard to jurisdictional claims in maps and in any institutional affiliations.

## Supporting information


**Dataset S1** Data shown in Fig. 2 and used to calibrate the soil‐plant hydraulic model for beech and spruce.

**Fig. S1** ‘WaldLab’ study site location and description.
**Fig. S2** Soil water retention curves of different soil samples from the study site.
**Fig. S3** Calibrated soil–plant hydraulic model on *E*_leaf_ and Ѱ_leaf_ measurements.
**Fig. S4** Validation of the soil–plant hydraulic model on sub‐daily Ѱ_leaf_ dynamics.
**Fig. S5** Atmospheric vapor pressure deficit and solar radiation during the two days used to validate the soil–plant hydraulic model in Fig. S4.
**Fig. S6** Timings of the maxima in sap flow *q*_s_ (*x*‐axis) and root water uptake (RWU) colored by the soil water content at 40 cm depth.
**Fig. S7** Timings of the maxima in sap flow *q*_s_ (*x*‐axis) and root water uptake (RWU) colored by the soil water content at 10 cm depth.
**Fig. S8** Timings of the maxima in sap flow *q*_s_ (*x*‐axis) and root water uptake (RWU) colored by the soil water content at 20 cm depth.
**Fig. S9** Timings of the maxima in sap flow *q*_s_ (*x*‐axis) and root water uptake (RWU) colored by the soil water content at 80 cm depth.
**Methods S1** VPD correction, calculation of leaf level transpiration *E*_leaf_ and model calibration.
**Methods S2** Granier‐type equation used to convert temperature differences to sap flow densities.
**Methods S3** Soil measurements.
**Methods S4** Soil–plant hydraulic model equations and parameters.
**Table S1** Soil–plant hydraulic model parameters.
**Table S2** Mean average errors (MAE) from the calibration and validation on individual trees.
**Table S3** Measured capacitances on root segments.
Please note: Wiley is not responsible for the content or functionality of any Supporting Information supplied by the authors. Any queries (other than missing material) should be directed to the *New Phytologist* Central Office.

## Data Availability

Data available in article Supporting Information (Dataset S1). The model code used to simulate soil–plant hydraulics is available under https://gitlab.com/SteveMacSteve/soil_plant_hydraulics_capacitance.
