# Supplementary material for: The interplay between hydraulic capacitance and stomatal regulation strategy affects soil–plant hydraulics and transpiration
Source: New Phytol. 2026 Mar 30;250(5):2988–3000. doi: 10.1111/nph.71143 (PMC13150304; doi:10.1111/nph.71143)
Supplement: Supplementary file 2 — Fig. S1 ‘WaldLab’ study site location and description. Fig. S2 Soil water retention curves of different soil samples from the study site. Fig. S3 Calibrated soil–plant hydraulic model on E leaf and Ѱleaf measurements. Fig. S4 Validation of the soil–plant hydraulic model on sub‐daily Ѱleaf dynamics. Fig. S5 Atmospheric vapor pressure deficit and solar radiation during the two days used to validate the soil–plant hydraulic model in Fig. S4. Fig. S6 Timings of the maxima in sap flow q s (x‐axis) and root water uptake (RWU) colored by the soil water content at 40 cm depth. Fig. S7 Timings of the maxima in sap flow q s (x‐axis) and root water uptake (RWU) colored by the soil water content at 10 cm depth. Fig. S8 Timings of the maxima in sap flow q s (x‐axis) and root water uptake (RWU) colored by the soil water content at 20 cm depth. Fig. S9 Timings of the maxima in sap flow q s (x‐axis) and root water uptake (RWU) colored by the soil water content at 80 cm depth. Methods S1 VPD correction, calculation of leaf level transpiration E leaf and model calibration. Methods S2 Granier‐type equation used to convert temperature differences to sap flow densities. Methods S3 Soil measurements. Methods S4 Soil–plant hydraulic model equations and parameters. Table S1 Soil–plant hydraulic model parameters. Table S2 Mean average errors (MAE) from the calibration and validation on individual trees. Table S3 Measured capacitances on root segments. Please note: Wiley is not responsible for the content or functionality of any Supporting Information supplied by the authors. Any queries (other than missing material) should be directed to the New Phytologist Central Office. [file NPH-250-2988-s002.docx]

## *New Phytologist* Supporting Information

Article title: The interplay between hydraulic capacitance and stomatal regulation strategy affects soil-plant hydraulics and transpiration

Authors: Stefano Martinetti, Andrea Carminati, Peter Molnar and Marius G. Floriancic

Article acceptance date: 01 March 2026

The following Supporting Information is available for this article:

Figure S1”WaldLab” study site location and description.

Figure S2 Soil water retention curves of different soil samples from the study site.

Figure S3 Calibrated soil-plant hydraulic model on E_leaf_ and Ѱ_leaf_ measurements.

Figure S4 Validation of the soil-plant hydraulic model on sub-daily Ѱ_leaf_ dynamics.

Figure S5 Atmospheric vapor pressure deficit and solar radation during the two days used to validate the soil-plant hydraulic model in Fig. S4.

Figure S6 Timings of the maxima in sap flow q_s_ (x-axis) and root water uptake (RWU) colored by the soil water content at 40 cm depth.

Figure S7 Timings of the maxima in sap flow q_s_ (x-axis) and root water uptake (RWU) colored by the soil water content at 10 cm depth.

Figure S8 Timings of the maxima in sap flow q_s_ (x-axis) and root water uptake (RWU) colored by the soil water content at 20 cm depth.

Figure S9 Timings of the maxima in sap flow q_s_ (x-axis) and root water uptake (RWU) colored by the soil water content at 80 cm depth.

Table S1 Soil-plant hydraulic model parameters.

Table S2 Mean average errors (MAE) from the calibration and validation on individual trees.

Table S3 Measured capacitances on root segments.

Methods S1 VPD correction, calculation of leaf level transpiration Eleaf and model calibration.

Methods S2 Granier-type equation used to convert temperature differences to sap flow densities.

Methods S3 Soil measurements.

Methods S4 Soil-plant hydraulic model equations and parameters.

**Supplementary Figures**


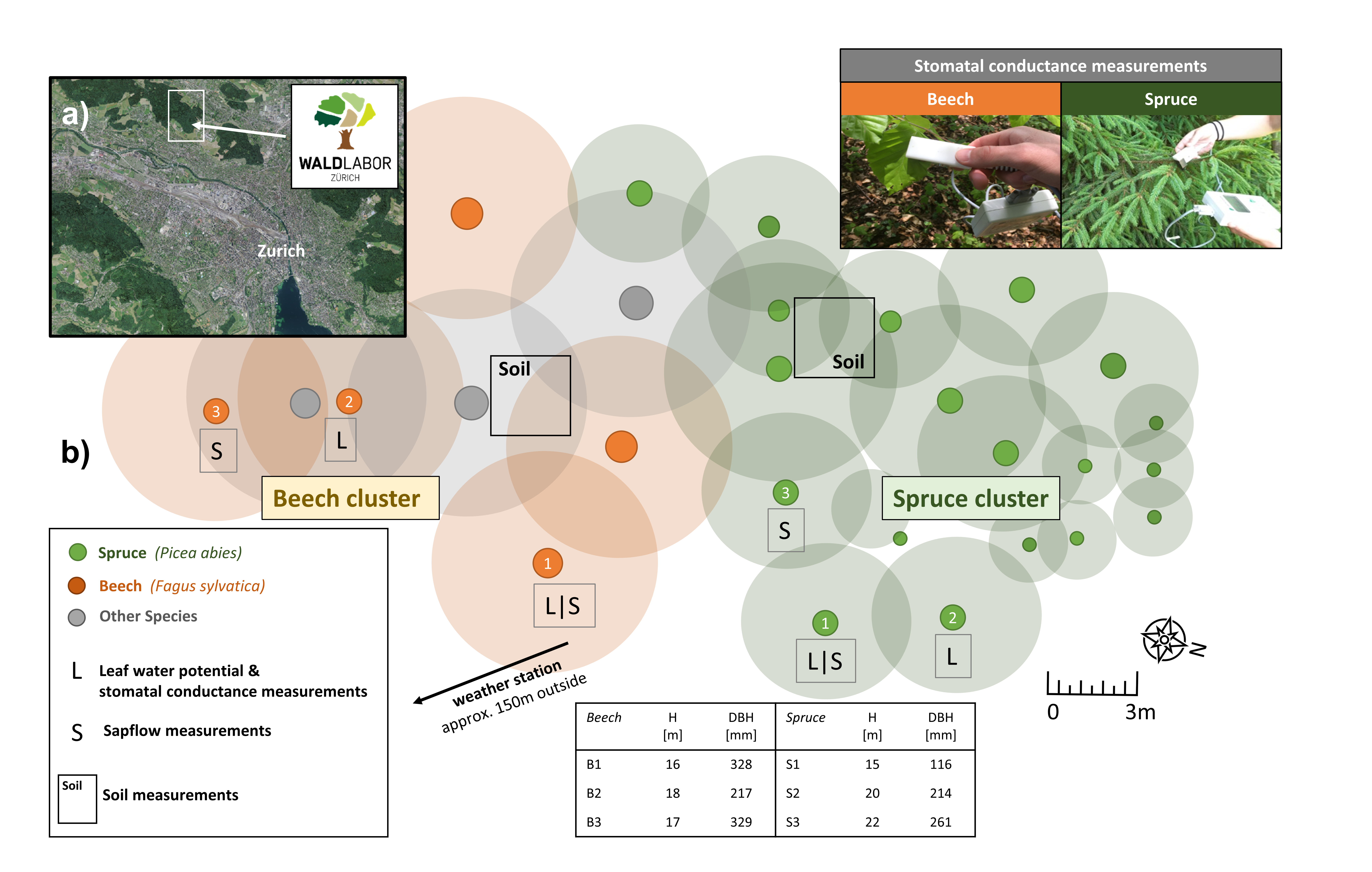


**Fig. S1** Location of the ‘WaldLab’ study site in Zurich (a) and a schematic of the experimental site (b), indicating the locations of trees (spruce, beech and other species shown in green, orange and grey). The trees where stem sap flow measurements were taken are marked with S, the trees where leaf-scale measurements were taken are marked with L. The location of the soil water measurements is indicated by a black box. The weather station and precipitation collector are located outside the forest, approximately 150 m from the experimental site. Tree heights (H) and diameters at breast height (DBH) of trees used for measurements are listed in the table in the lower center.


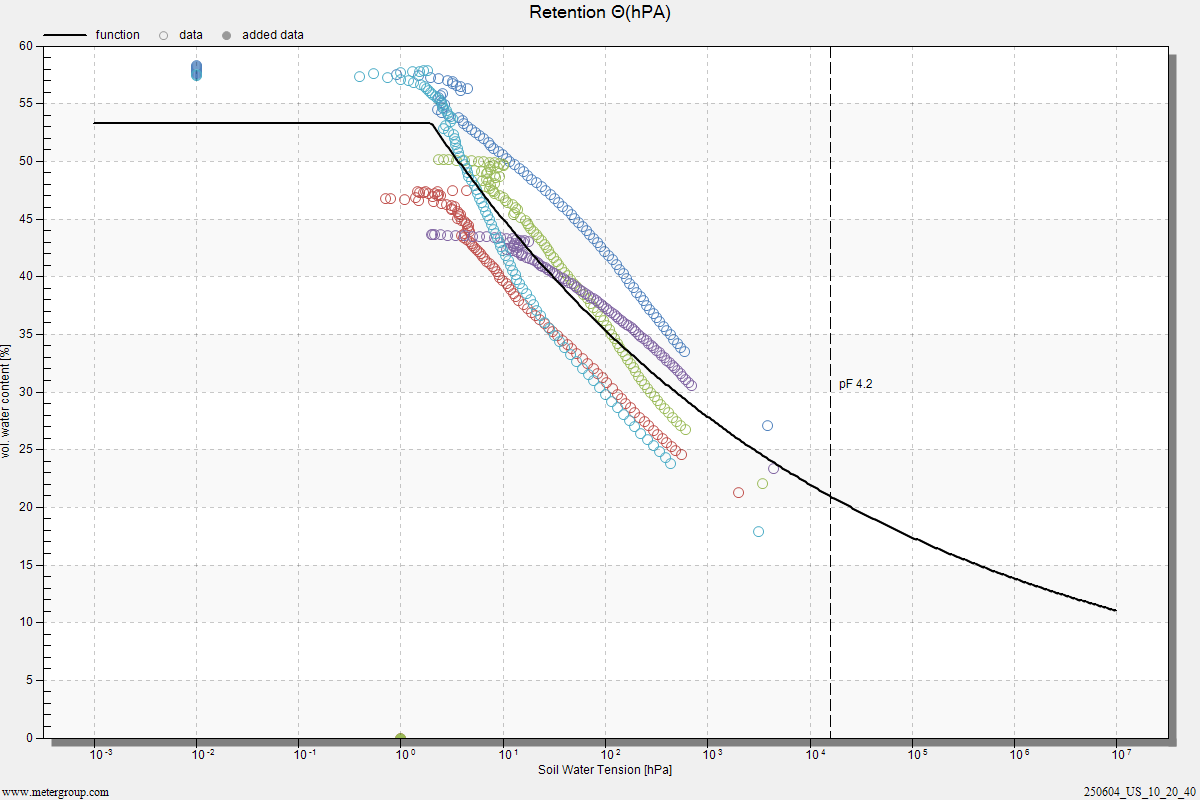


**Fig. S2** Soil retention curve obtained from Hyprop measurements at 10 cm (beech – blue, spruce – light blue), 20 cm (beech – red, spruce – green) and 40 cm (violet). The figure was produced by the LABROS SoilView-Analysis version 5.1.1.0 software and with fitted parameters listed in Table S1.


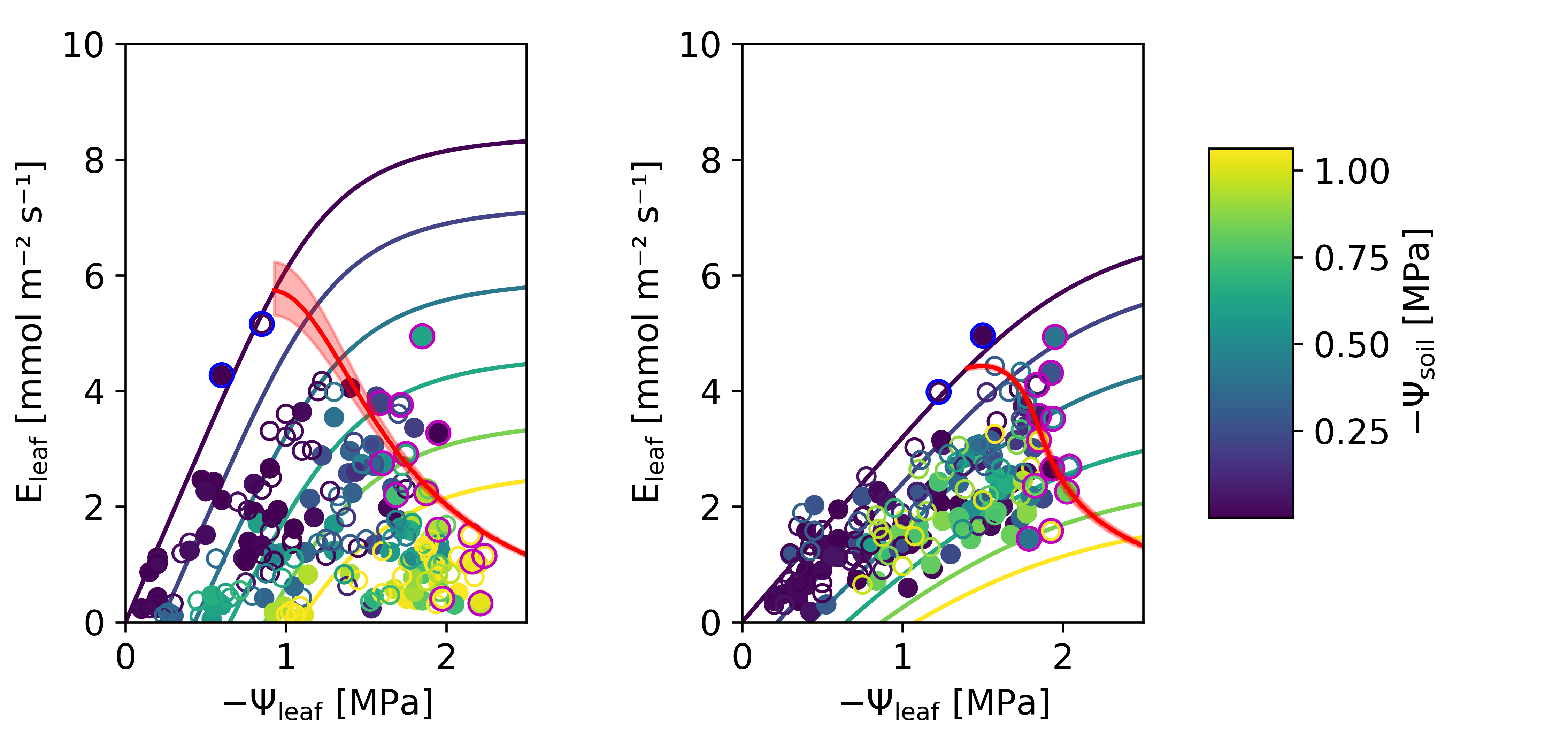


**Fig. S3** Calibrated soil-plant hydraulic model on leaf transpiration E_leaf_ measurements derived from stomatal conductance and VPD measured at the leaf. The different trees where stomatal conductance and leaf water potential were measured are indicated by filled and empty circles. The model parameters are listed in Table S1. Data points with a red edge were used to estimate the root length L and the onset of loss of hydraulic conductivity h_x0_ by comparing measurements (dots with red edge) to simulations (red curve). The data points with a blue edge were used to calibrate max. soil-plant hydraulic conductance K_x0_. Shading indicates the parameterization obtained from individual trees, which were averaged to obtain a species-specific parameterizations. Calibration and validation mean absolute errors are listed in Table S2.


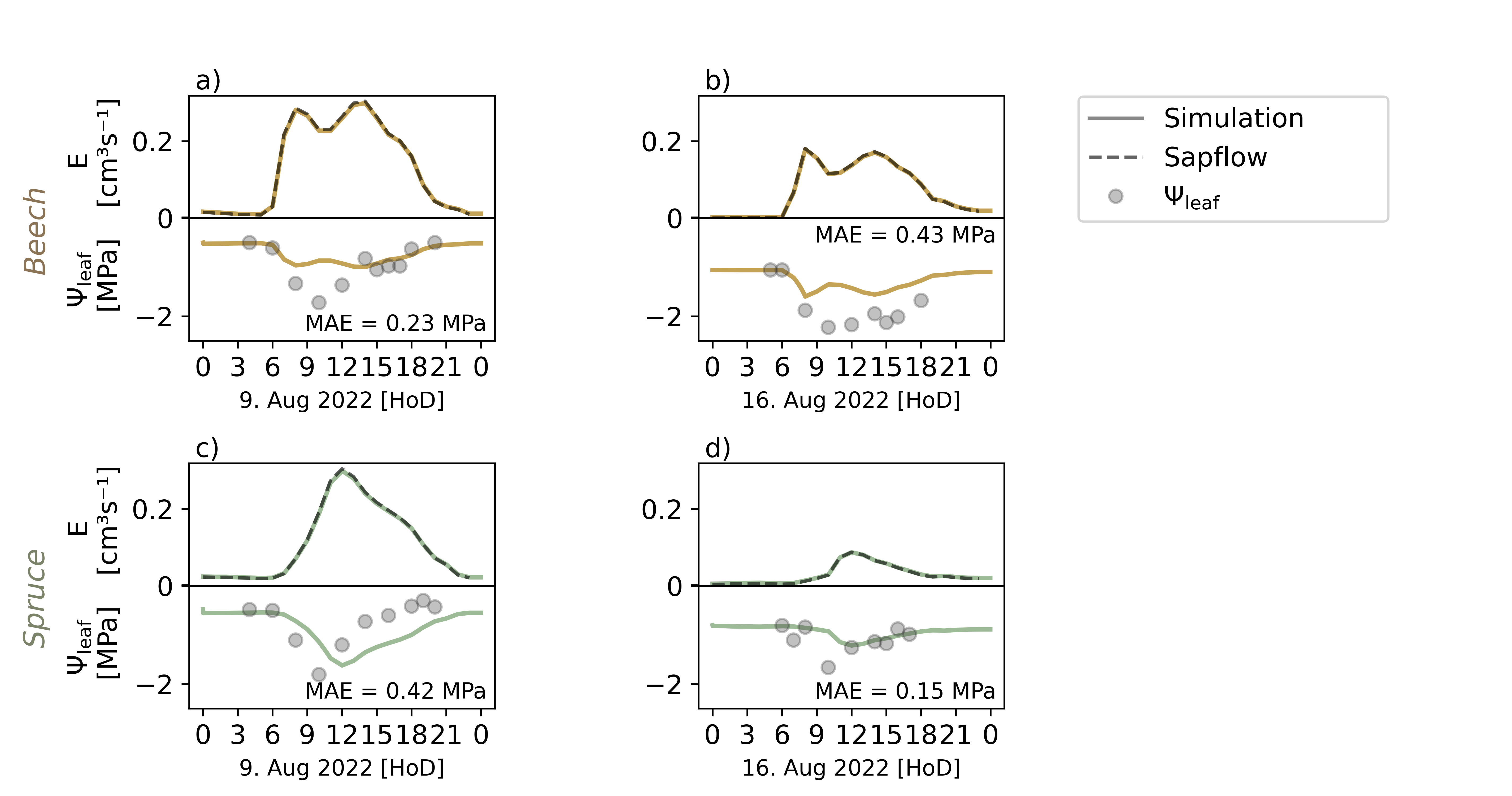


**Fig. S4** Simulations and observations of leaf water potentials on two days with similar atmospheric conditions, but different soil water contents. The simulations were performed with sap flow, rescaled to a maximum of 0.3 cm^3^s^-1^ in order to match the maximum evaporative demand used in Fig. **4**, as the transpiration boundary condition, and the measured pre-dawn leaf water potentials as the initial soil water potentials. Mean absolute error (MAE) between observed and simulated leaf water potentials are reported in each panel. The simulations capture the general dynamics in leaf water potential, but have relatively high MAEs. However, the models boundary condition (i.e., evaporative demand) is determined by sap flow dynamics, which due to capacitance further upstream the location of the sensor could underestimate true transpiration, thereby leading to an overestimation of leaf water potentials. Also, the single-leaf approach employed in the model does not consider heterogeneities in leaf water potential across the canopy, which our measurements are affected by.


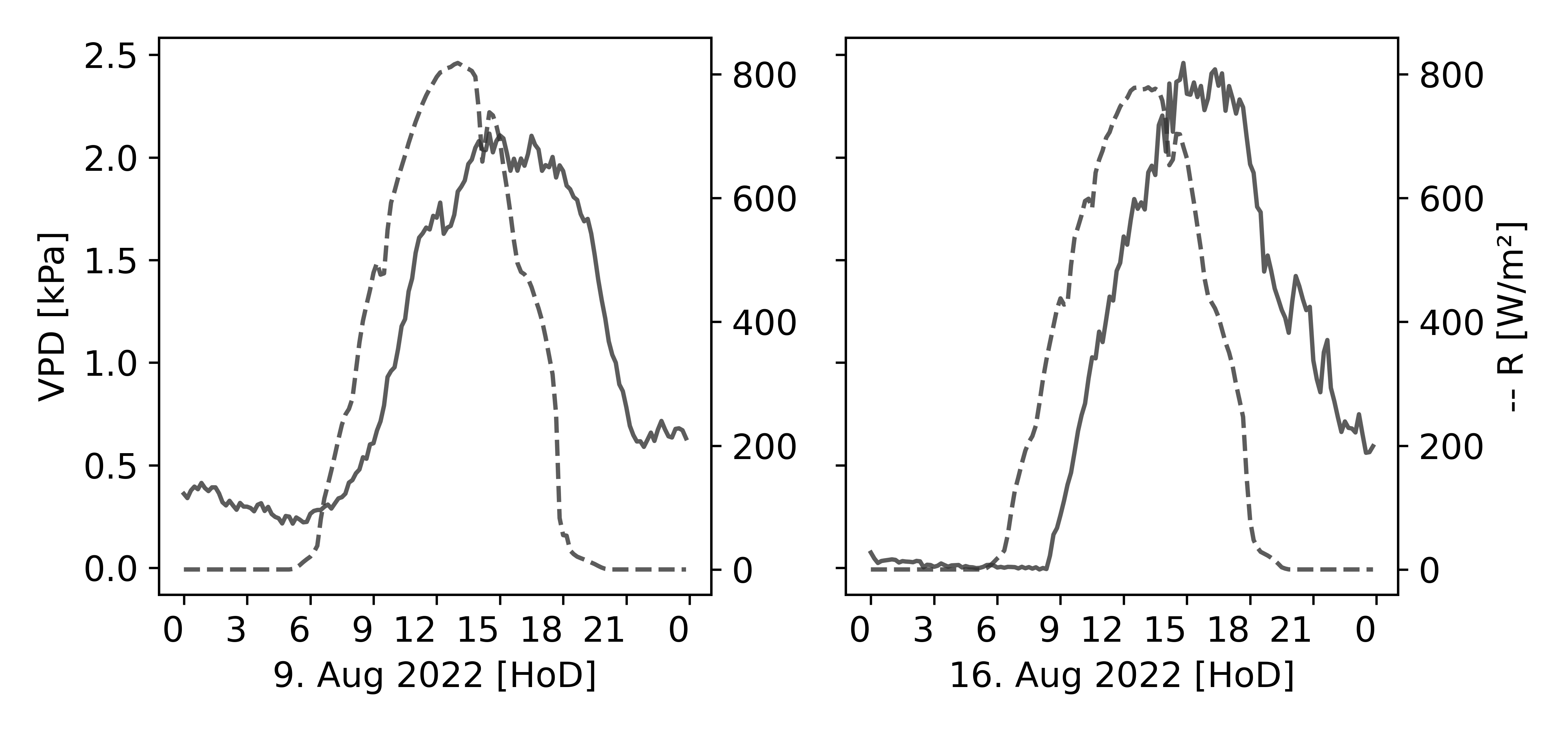


**Fig. S5** Vapor pressure deficit (VPD – solid line) and solar radiation (R – dashed line) measured during the two days shown in Fig. S4 (9^th^ and 16^th^ August 2022). Both variables were measured at the weather station near our field site (see Fig. S1). Both days were sunny and warm.


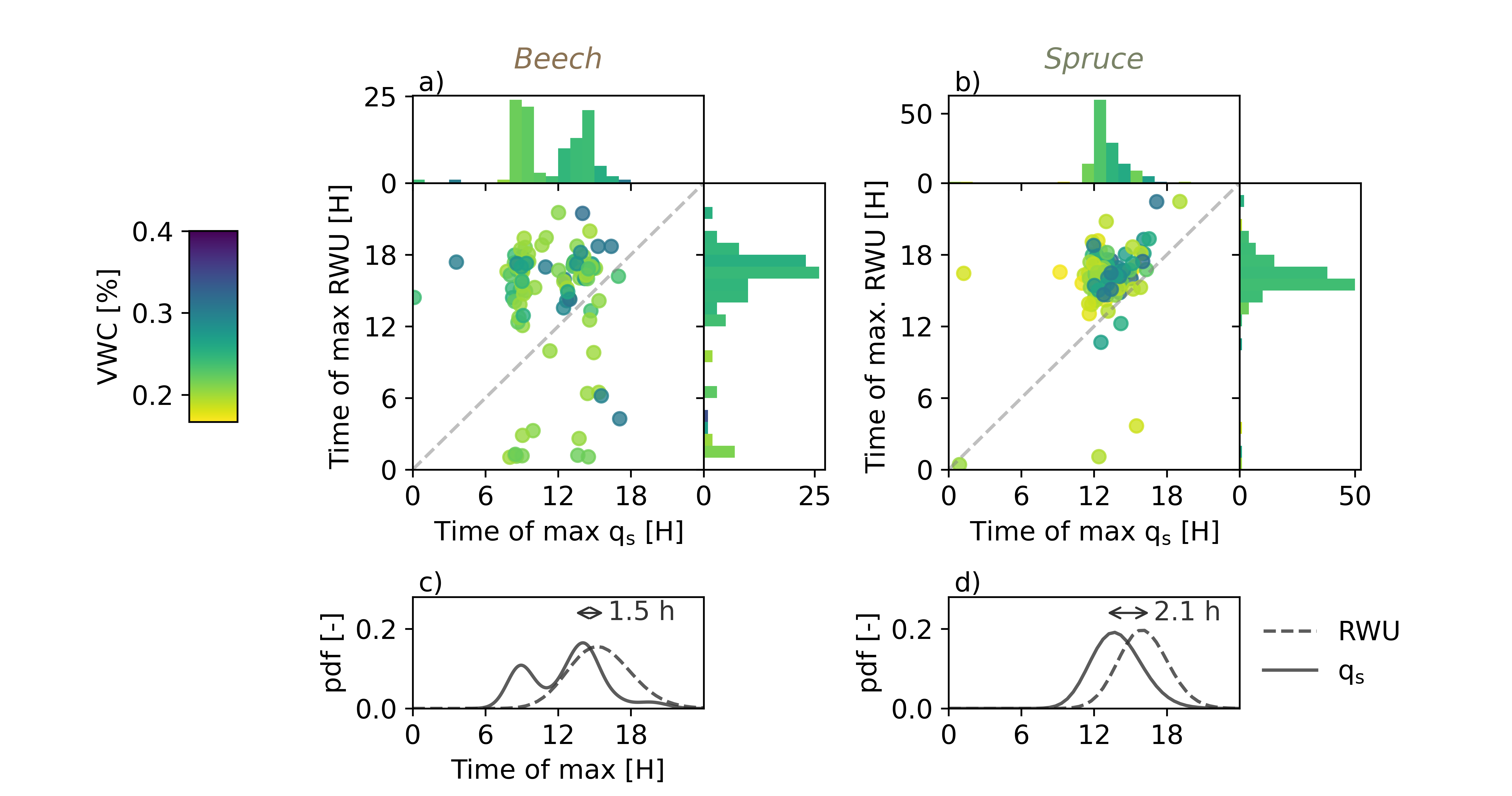


**Fig. S6** The timings of the maxima in sap flow q_s_ (x-axis) and root water uptake (RWU) estimated from soil water content at 40 cm depth (y-axis) for beech (a – left panel) and spruce (b- right panel), along with their respective histograms. The color indicates the soil water content at 40 cm depth. Fitted probability density distributions (c&d) show mean time lags between maxima of sap flow and root water uptake, resulting from capacitance. The upper row shows data of days when both, sap flow and root water uptake could be computed, while probability density functions (gamma distribution for root water uptake and spruce sap flow and kernel density estimation for beech sap flow) in the middle row were estimated from all data obtained during the growing seasons (April-September) of 2022 through 2024. Spruce showed larger time lags between daily peaks in sap flow and root water uptake suggesting larger capacitance.


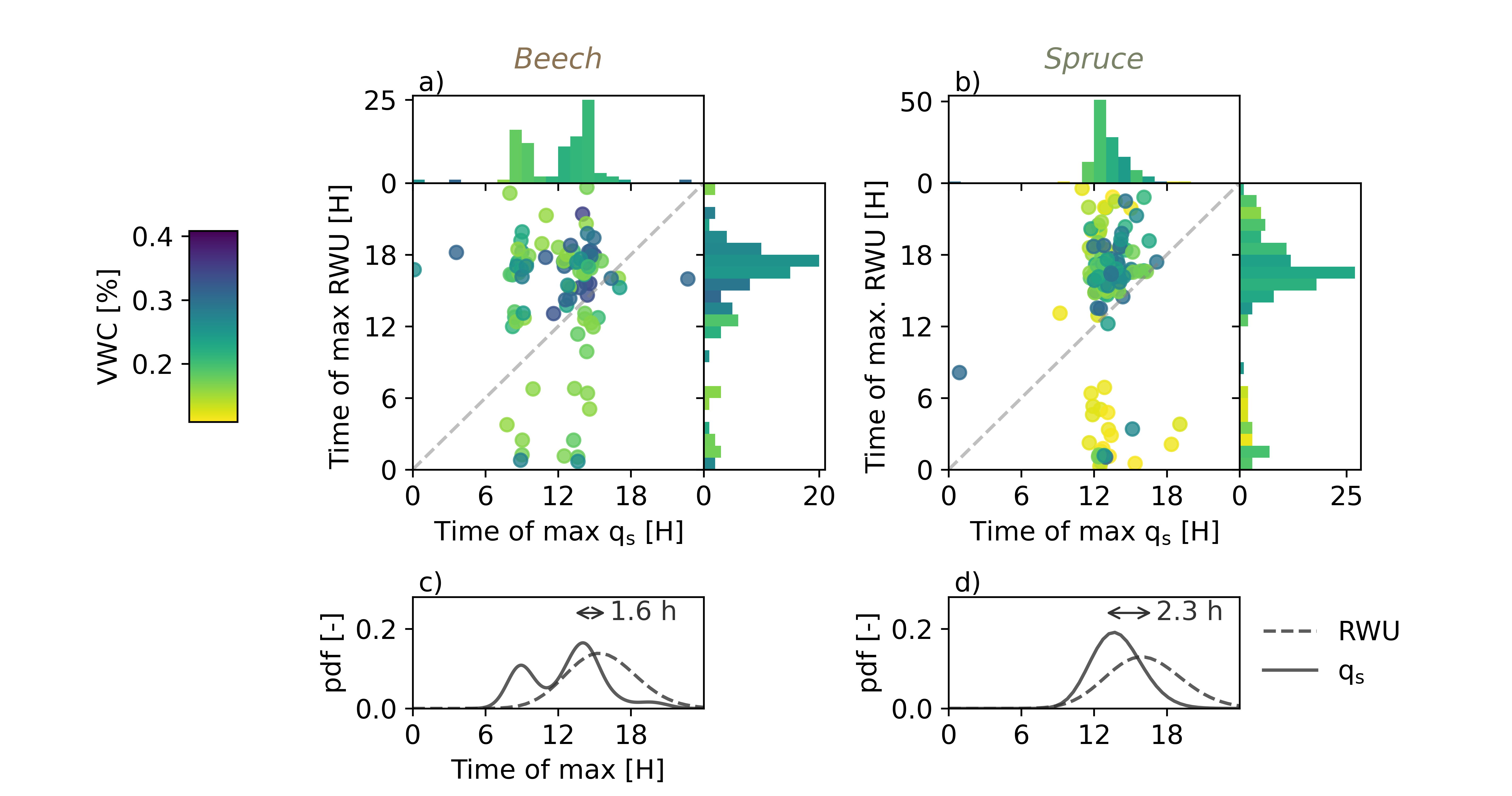


**Fig. S7** The timings of the maxima in sap flow q_s_ (x-axis) and root water uptake (RWU) estimated from soil water content at 10 cm depth (y-axis) for beech (a – left panel) and spruce (b- right panel), along with their respective histograms. The color indicates the soil water content at 10 cm depth. Fitted probability density distributions (c&d) show mean time lags between maxima of sap flow and root water uptake, resulting from capacitance. The upper row shows data of days when both, sap flow and root water uptake could be computed, while probability density functions (gamma distribution for root water uptake and spruce sap flow and kernel density estimation for beech sap flow) in the middle row were estimated from all data obtained during the growing seasons (April-September) of 2022 through 2024. Spruce showed larger time lags between daily peaks in sap flow and root water uptake suggesting larger capacitance.


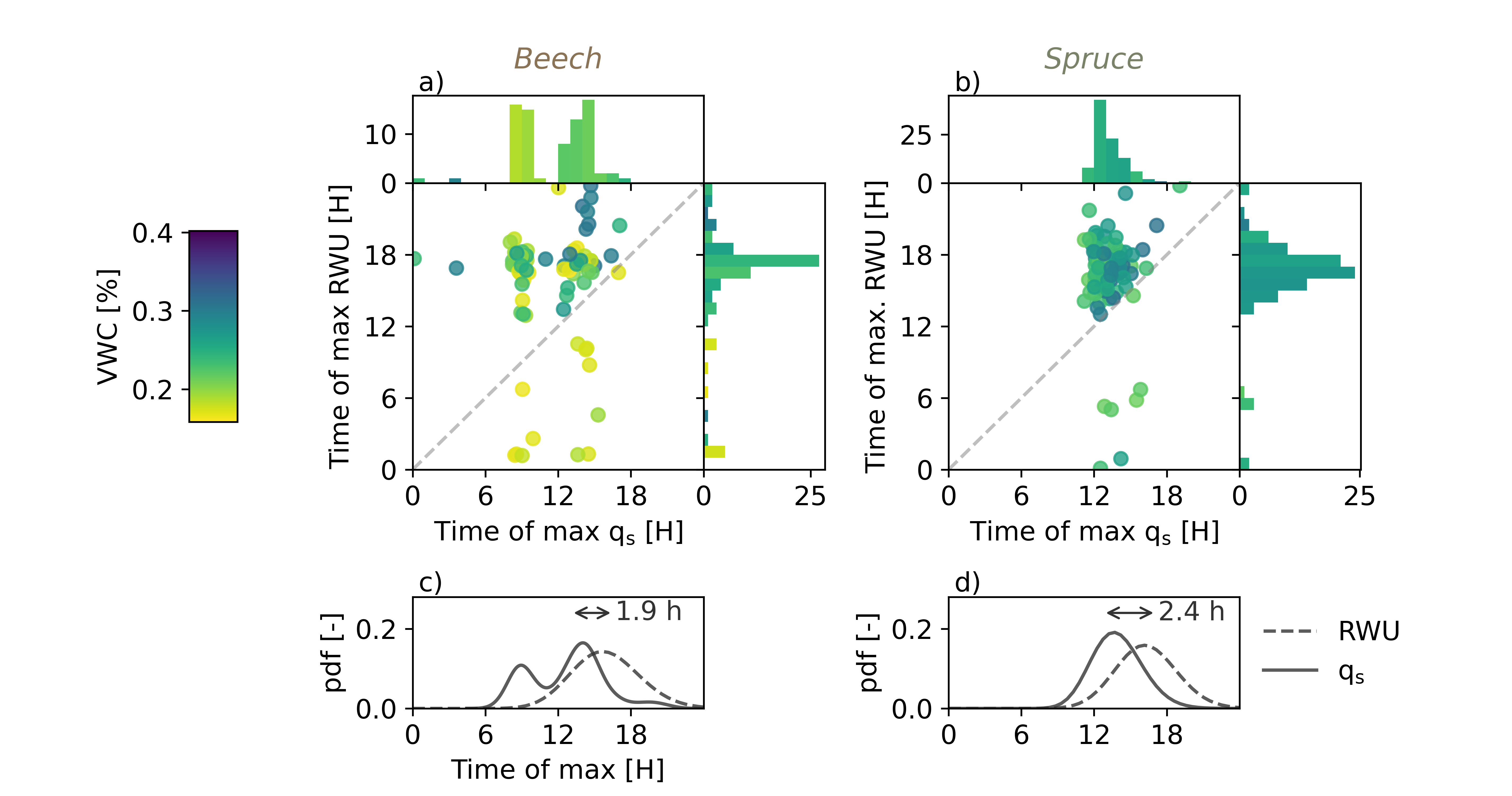


**Fig. S8** The timings of the maxima in sap flow q_s_ (x-axis) and root water uptake (RWU) estimated from soil water content at 20 cm depth (y-axis) for beech (a – left panel) and spruce (b- right panel), along with their respective histograms. The color indicates the soil water content at 20 cm depth. Fitted probability density distributions (c&d) show mean time lags between maxima of sap flow and root water uptake, resulting from capacitance. The upper row shows data of days when both, sap flow and root water uptake could be computed, while probability density functions (gamma distribution for root water uptake and spruce sap flow and kernel density estimation for beech sap flow) in the middle row were estimated from all data obtained during the growing seasons (April-September) of 2022 through 2024. Spruce showed larger time lags between daily peaks in sap flow and root water uptake suggesting larger capacitance.


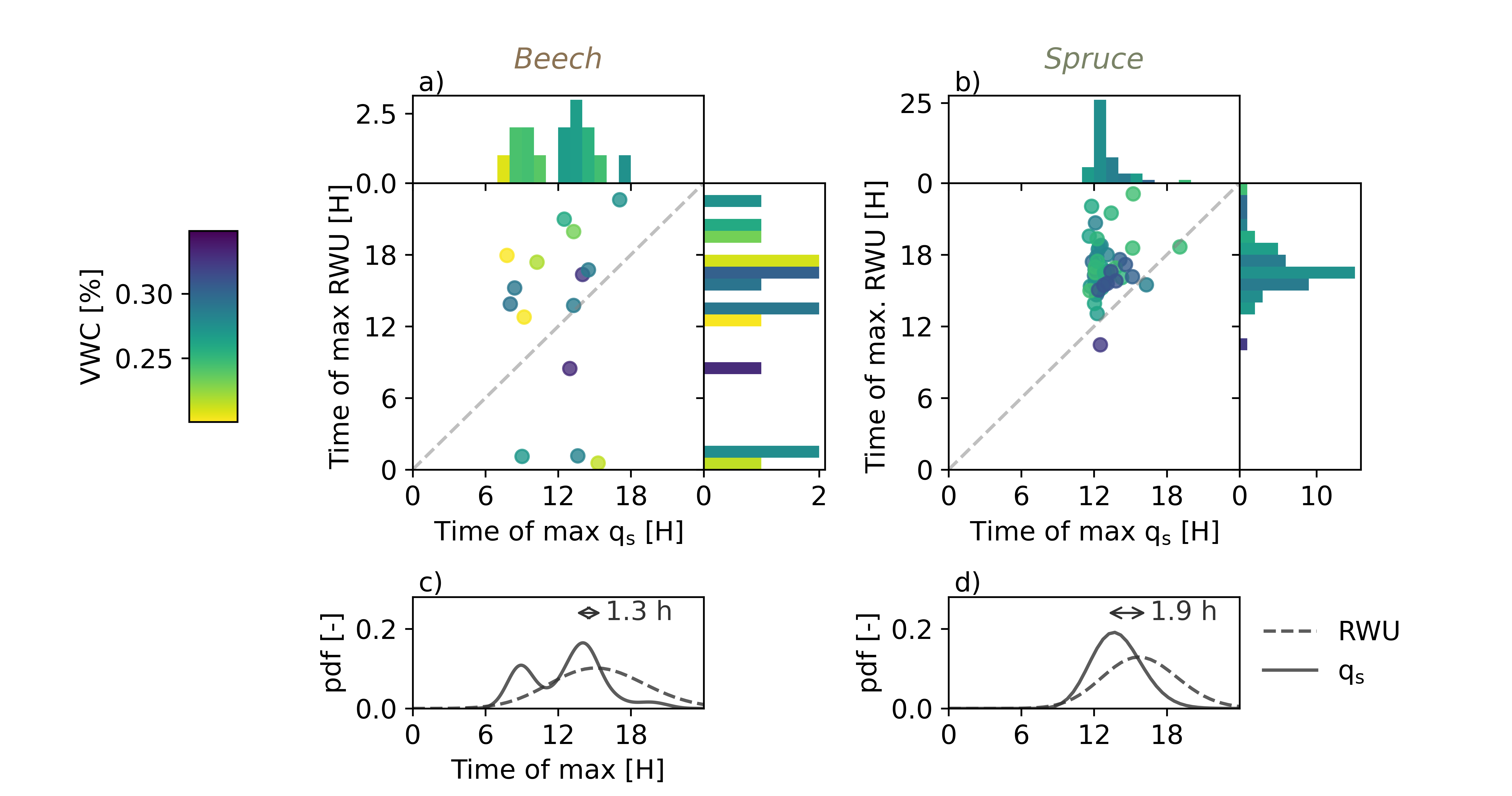


**Fig. S9** The timings of the maxima in sap flow q_s_ (x-axis) and root water uptake (RWU) estimated from soil water content at 80 cm depth (y-axis) for beech (a – left panel) and spruce (b- right panel), along with their respective histograms. The color indicates the soil water content at 80 cm depth. Fitted probability density distributions (c&d) show mean time lags between maxima of sap flow and root water uptake, resulting from capacitance. The upper row shows data of days when both, sap flow and root water uptake could be computed, while probability density functions (gamma distribution for root water uptake and spruce sap flow and kernel density estimation for beech sap flow) in the middle row were estimated from all data obtained during the growing seasons (April-September) of 2022 through 2024. Spruce showed larger time lags between daily peaks in sap flow and root water uptake suggesting larger capacitance.

Click here to enter text.

**Supplementary Tables**

**Table S1** **Parameters selected from literature (Abdalla et al. 2022, Wankmüller et al. 2022) (in italic) and calibrated based on measurements (in plain text) for the soil-plant hydraulic simulations.**

| **Parameter** | **Value** | **Unit** |
| --- | --- | --- |
| Max. soil-plant hydraulic conductance K_x0_ | \| 0.77 \| *B1* \| \| --- \| --- \| \| 0.66 \| *B2* \| \| 0.36 \| *S1* \| \| 0.35 \| *S2* \| | cm^3^ (MPa s)^-1^ |
| Root length L | \| 25500 \| *B1* \| \| --- \| --- \| \| 29250 \| *B2* \| \| 10500 \| *S1* \| \| 9750 \| *S2* \| | m |
| Xylem water potential at onset of loss of conductivity h_x0_ | \| -1.7 \| *B1* \| \| --- \| --- \| \| -1.7 \| *B2* \| \| -2.8 \| *S1* \| \| -2.8 \| *S2* \| | MPa |
| Accessible soil volume V_s_ | 1/3 | m^3^ |
| Leaf Area *LA* | *6* | m^2^ |
| Brooks-Corey exponent Xylem τ_x_ | *5* | - |
| Saturated soil hydraulic conductivity k_s_ | 53.6 | cm d^-1^ |
| Soil water potential at onset of loss of conductivity h_0_ | -2 | cm |
| Brooks-Corey exponent Soil τ | 2.112 | - |
| Saturated soil water content | 0.533 | m^3^ m^-3^ |
| Residual soil water content | 0.027 | m^3^ m^-3^ |
| Brooks-Corey α | 0.5 | cm^-1^ |
| Brooks-Corey λ | 0.112 | - |
| Root radius r_0_ | *0.5* | mm |
| Radius of soil surrounding the root r_b_ | 2.03 (Beech)  3.28 (Spruce) | mm |
| Water density ρ | *999.7* | Kg m^-3^ |
| Standard gravity g | *9.81* | m s^-2^ |

**Table S2 Calibration and validation mean average errors (MAE) in leaf water potential (Ѱ_leaf_) and leaf transpiration (E) for the predicted onset of stomatal closure determined by the soil-plant hydraulic model and the maximization strategy for individual trees and species-average parameterization (italic).**

| **Tree** | **Calibration MAE** | | **Validation MAE** | |
| --- | --- | --- | --- | --- |
|  | **Ѱ_leaf_ [MPa]** | **E [mmol m^2^ s^-1]^]** | **Ѱ_leaf_ [MPa]** | **E [mmol m^2^ s^-1]^]** |
| Beech 1 | 0.35 | 0.92 | 0.30 | 0.57 |
| Beech 2 | 0.31 | 0.58 | 0.36 | 0.89 |
| *Beech* |  |  | *0.35* | *0.81* |
| Spruce 1 | 0.25 | 1.07 | 0.16 | 0.76 |
| Spruce 2 | 0.15 | 0.75 | 0.24 | 1.12 |
| *Spruce* |  |  | *0.2* | *0.92* |

**Table S3 Capacitance estimates obtained on root segments by monitoring changes in mass (water) and xylem water potential. The capacitance estimates correspond to the slopes of the regression lines through the trajectories shown in Figure 3. Estimates in [kg MPa^-1^ kg^-1^] were obtained by considering the dry weight of the root segment, while estimates in [kg MPa^-1^ m^-3^] were obtained by considering a wood density of 400 kg m^-3^ and 700 kg m^-3^ for spruce and beech, respectively.**

| **Root** | **Dry weight [g]** | **[kg MPa^-1^ kg^-1^]** | | **[kg MPa^-1^ m^-3^]** |
| --- | --- | --- | --- | --- |
| Beech segment 1 | 52 | | 0.034 | 23.5 |
| Beech segment 2 | 47 | | 0.062 | 43.3 |
| Beech average |  | | 0.048 | 33.4 |
| Spruce segment 1 | 18 | | 0.162 | 64.7 |
| Spruce segment 2 | 28 | | 0.111 | 67.9 |
| Spruce average |  | | 0.136 | 66.3 |

**Supplementary Methods**

**Methods S1** **VPD correction, calculation of leaf level transpiration E_leaf_ and model calibration**

We measured temperature and relative humidity with SHT85 Sensors near the leaf where stomatal conductance and leaf water potential measurements were performed. These temperature and relative humidity readings were used to calculate VPD at the leaf. However, the sensor was not equipped with a radiation protection and was prone to overheating during measurements, especially when the sensor was exposed to radiation for long periods of time, causing some excessively high VPD readings. The excessively high manual VPD readings were determined by assuming a perfect fit between manual VPD and meteostation VPD, and calculating the 80% prediction band of the regression line of the meteostation VPD readings (i.e., 1-1 line). To correct for high VPD measurements at the leaf, all manual VPD measurements above the 80% prediction band were reduced such that they would lie at the 80% prediction band (see Fig. A).

The calculation of E_leaf_ assumes i) negligible aerodynamic boundary layer conductance of the thin layer of air around the leaves (i.e., conductance is high or resistance is low) and ii) negligible cuticular transpiration contributing to total E_leaf_. Aerodynamic boundary layer conductance was likely negligible during the stomatal conductance measurements. Using equation 3 in Meinzer *et al.* (1997) with characteristic leaf dimensions equal to 5 cm (for beech) and wind speeds of 0.2 m/s (minimum wind speed occurring at the meteostation during our measurements), estimates of aerodynamic conductances were above 400 mmol m 2 s-1. Therefore, measured stomatal conductances were consistently lower (stomatal resistances were consistently higher) than the aerodynamic boundary layer conductances (as higher wind speeds would even further increase aerodynamic conductances), supporting that stomatal conductance was the main control of leaf level transpiration. This method is not flawless, but E_leaf_ estimates here were only used to calibrate model parameters (Fig. S3) and do not constitute substantial results of this study.


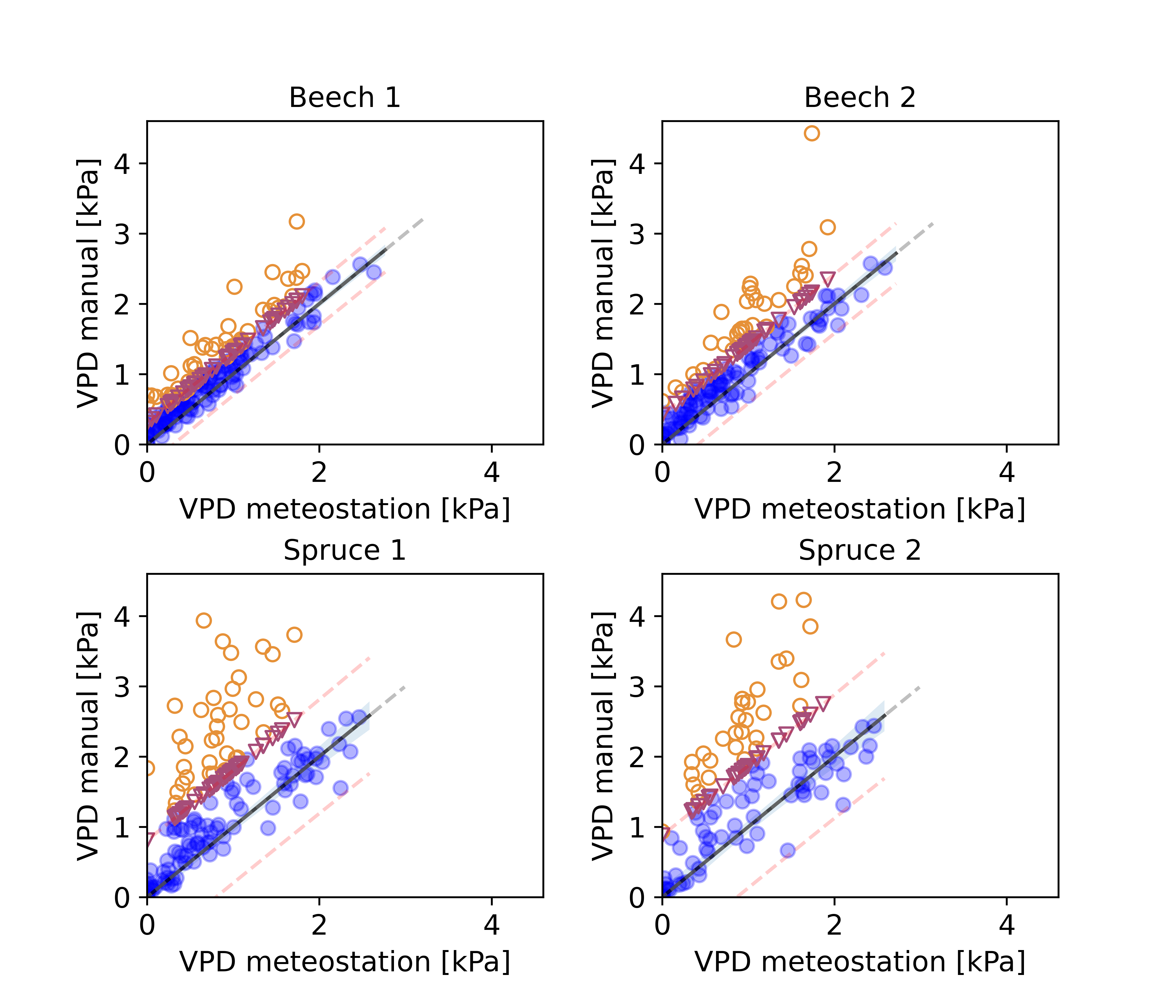


**Fig. A** Manual VPD measurements at the different trees performed during stomatal conductance measurements (y-axis) and corresponding measurements of VPD at the meteostation (x-axis). The linear regression between the meteostation VPD measurements only was used to correct excessively high manual VPD values (light brown circles) by reducing them such that they would lie on the 80% prediction band (dark brown triangles) of the regression.

**Methods S2** **Granier-type equation used to convert temperature differences to sap flow densities**

$$\begin{aligned} u={213.625*\left( \frac{{dT}_{max}}{{dT}_{actual}}-1 \right)}^{1.509}=213.625*k^{1.509} \#S1 \end{aligned}$$

where is *u* the sap flow density in *cm^3^ m^-2^ s⁻¹*, *dT_max_* is the temperature difference occurring under zero-flow conditions (at predawn), calculated over a moving window of 5 days, and *dT_actual_* is the current temperature difference. We obtained calibrated parameters from fitting the equation of the form of Eq.  S1 to experimental data from beech and spruce samples (Peters *et al.*, 2021) in Fig. B.


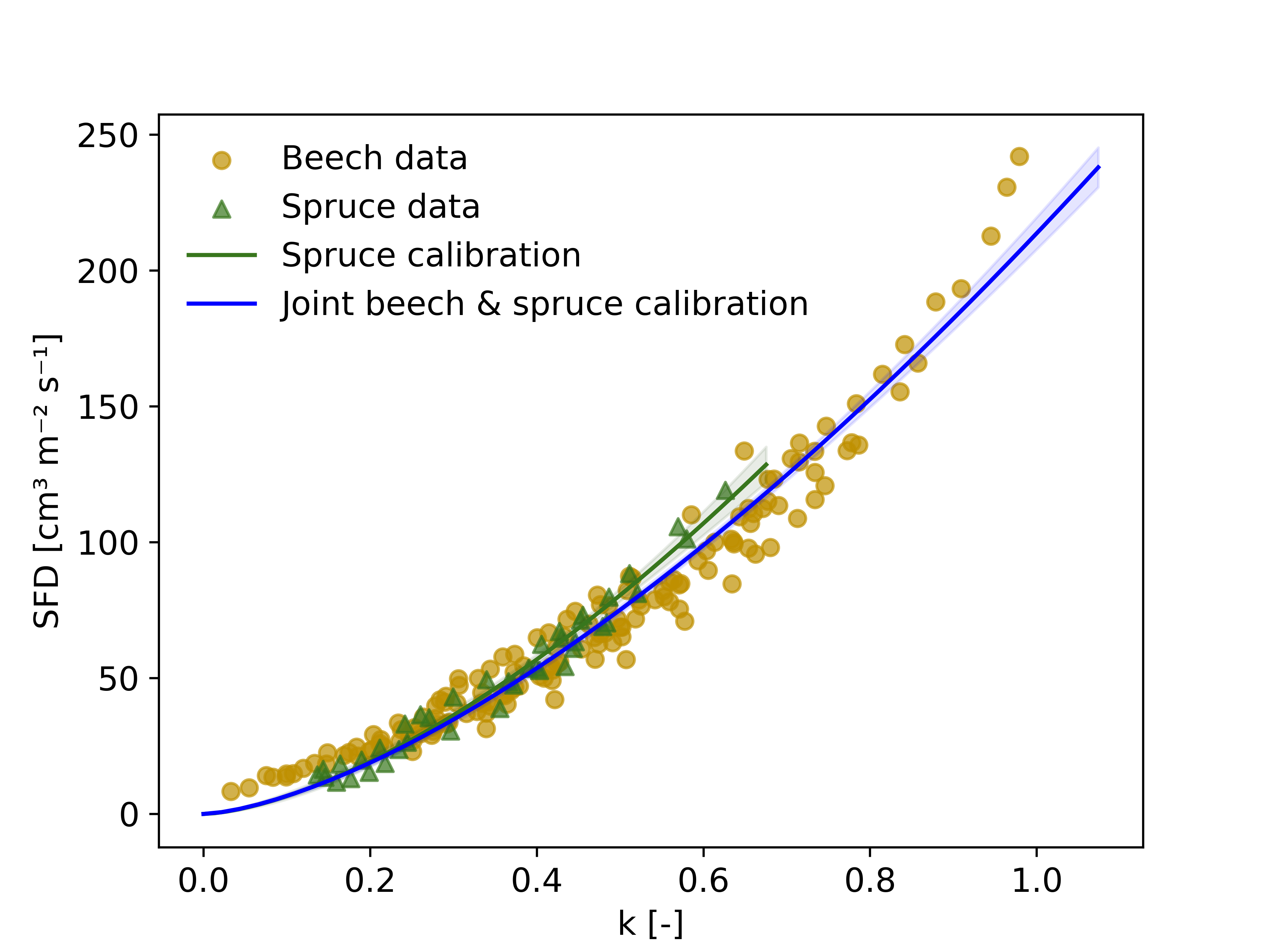


**Fig. B** Calibration function (Eq. S1) used to convert temperature differences (k, see Eq. S1) measured with sap flow sensors into sap flow densities (SFD). The brown circles and green triangles show data from Peters *et al.* (2021) of beech and spruce, respectively. The blue curve shows the joint calibration for beech and spruce data together, while the green curve shows the calibration for spruce data only. The shading around the curves show the estimated 95% confidence of the fitted parameters. There is no strong difference between the two species, which is why we opted to use the same joint calibration for both species.

**Methods S3** **Soil measurements**

Saturated hydraulic conductivities (Fig. C) were measured with a permeameter (Royal Eijkelkamp), and soil water retention curves (Fig. S2) and unsaturated hydraulic conductivities (Fig. D) were measured with the Hyprop 2 (Meter AG) device at 10, 20 and 40 cm (only Hyprop 2 measurements at 40 cm depth). We digged a hole at the desired depth, once within the beech and once within the spruce cluster, and sampled the soil three times at each depth with a cylinder for the permeameter (5.6 cm in diameter and 4.05 cm in height) and once with the cylinder for the Hyprop (250 ml of soil). At 40 cm depth we only sampled near the spruce cluster. The probes were then brought to the lab, and saturated in a water filled tank as described by the manufacturer. The permeameter probes were subsequently put into the permeameter body. We then measured the saturated hydraulic conductivity by keeping the water head constant and measuring the volume of water passing through the sample during measured time intervals, and repeated the measurement procedure up to 8 times for each sample. The hyprop measurement was set up as described by the manufacturer and the measurement was stopped once the air entry point was reached. These soil measurements were mainly performed to obtain a representative soil parameterization of the site for the soil-plant hydraulic model. Parameters for the Brooks-Corey parameterization of soil were obtained clustering all hyprop measurements together, in order to obtain average soil-properties, and fitting the Brooks-Corey parameterization with the SoilView Software. Permeameter measurements of saturated hydraulic conductivity were highly variable, but averaged around 50 cm d-1, therefore the measured saturated hydraulic conductivity is matching the fitted saturated hydraulic conductivity of 53.6 cm d^-1^.


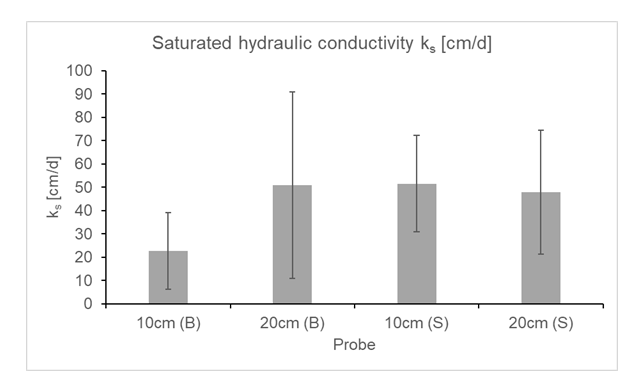


**Fig. C** Saturated hydraulic conductivity k_s_ of the soil at different depths. These results were obtained from three soil probes at 10 and 20 cm depth near beech (B) and spruce (S) trees, totaling 12 probes. We performed eight measurements per probe and here show average and standard deviation of the last four measurements of all soil probes at the probing sites.


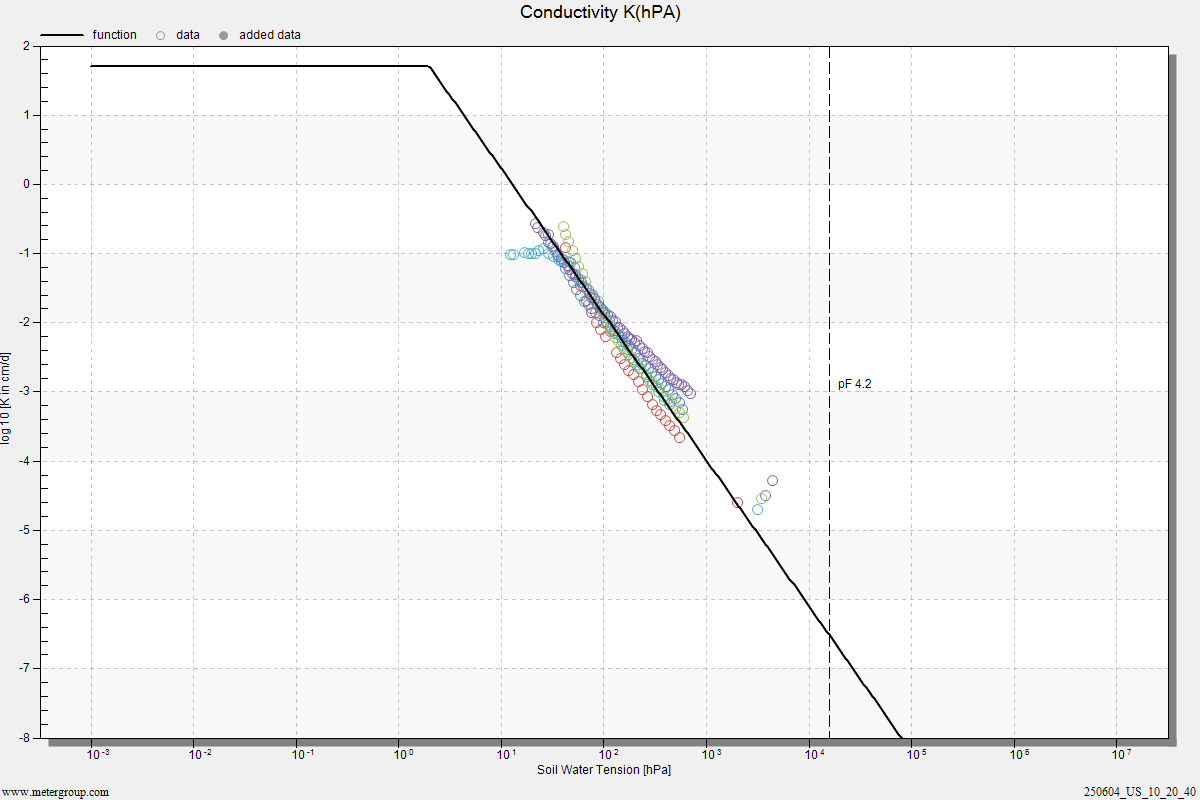


**Fig. D** Soil hydraulic conductivity as a function of soil water potential obtained from Hyprop measurements at 10 cm (beech – blue, spruce – light blue), 20 cm (beech – red, spruce – green) and 40 cm (violet). The figure was produced by the LABROS SoilView-Analysis version 5.1.1.0 software and with fitted parameters listed in Table S1.

**Methods S4** **Soil-plant hydraulic model equations and parameters**

At each time step, the model iteratively solves for Ѱ_x_ that equilibrate the water flux from root water uptake Q_soil_, the water flux from plant water storage Q_cap_ and transpiration E. The water flux from plant water storage Q_cap_ is first calculated based on the water potential of the plant water storage Ѱ_cap_ and water potential in the xylem Ѱ_x_. Ѱ_cap_ is known from the previous time step, while Ѱ_x_ is either set equal to Ѱ_x_ of the previous time step (if it is the first iteration), or was obtained in the previous iteration.

$$\begin{aligned} Q_{cap}=K_{x0}\cdot\left( \Psi_{cap}-\Psi_{x} \right) \#S2 \end{aligned}$$

The water flux from the plant water storage Q_cap_ is subtracted from the imposed transpiration E to obtain the water flux from root water uptake Q_soil_.

$$\begin{aligned} Q_{soil}=E-Q_{cap} \#S3 \end{aligned}$$

The water flux from root water uptake Q_soil_ along with the water potential of the soil of the previous time step Ѱ_soil_ result in the water potential at the soil-root interface by solving the Richards equation for radial flux to the root and using matrix flux potential formulation along with the Brooks-Corey parameterization of unsaturated hydraulic conductivity (see also Wankmüller and Carminati 2022; Carminati and Javaux 2020).

$$\begin{aligned} \Psi_{soil-root}=-\left| \left| \Psi_{soil} \right|^{1-\tau} - \frac{Q_{soil}\left( 1-\tau\right)}{2\pi LK_{s0}\left| h_{*} \right|^{\tau}}\left( \frac{1}{2}-\frac{r_{b}^{2}\ln\left( \frac{r_{b}}{r_{0}} \right)}{r_{b}^{2}-r_{0}^{2}} \right) \right|^{\frac{1}{1-\tau}} \#S4 \end{aligned}$$

The water potential at the soil-root interface Ѱ_soil-root_ and the root water uptake Q_soil_ are subsequently used to calculate the xylem water potential Ѱ_x_’ resulting from root water uptake Q_soil_ from the root surface to the xylem.

$$\begin{aligned} \Psi_{x}^{'}=\psi_{soil-root}-K_{x0}\cdot Q_{soil}\#S5 \end{aligned}$$

This Ѱ_x_’ is averaged with the previous Ѱ_x_ to obtain the xylem water potential for the next iteration step Ѱ_x,new_. Once the difference between Ѱ_x_ and Ѱ_x,new_ is smaller than a set threshold (here 0.5 cm), the iteration is terminated, the respective water fluxes Q_soil_ and Q_cap_ are subtracted from soil and plant water storage, respectively, and the water potentials Ѱ_soil_ and Ѱ_cap_ for the next time step are calculated based on the water retention curve of the soil (obtained from Hyprop measurements and parameterized with Brooks-Corey) and the used capacitance C (obtained by setting plant water storage capacity and assuming that the storage linearly drains to 0 at a water potential of -4.5 MPa, Eq. S6).

$$\begin{aligned} C=\frac{dV_{cap}}{d\psi_{cap}}=\frac{\Delta V_{cap}}{\Delta\psi_{cap}}=\frac{V_{cap,max}}{-4.5 MPa}\#S6 \end{aligned}$$

$$\begin{aligned} \psi_{cap}=f(V_{cap}){=\frac{1}{C}\cdot(V}_{cap,max}-V_{cap})=-4.5 MPa\cdot(1-\frac{V_{cap}}{V_{cap,max}})\#S7 \end{aligned}$$

Then, the leaf water potential Ѱ_leaf_ is calculated based on the found xylem water potential Ѱ_x_ and the imposed transpiration E.

$$\begin{aligned} \Psi_{leaf}=-\left| \left| \Psi_{x} \right|^{1-\tau_{x}} + \frac{E\left( 1-\tau_{x} \right)}{K_{x0}\left| h_{x0} \right|^{\tau_{x}}} \right|^{\frac{1}{1-\tau_{x}}}\#S8 \end{aligned}$$

For the water fluxes Q_cap_ and Q_soil_ to the xylem (Eq. S2 and Eq. S5) a constant conductance K_x0_ is used, implying that the conductance for these fluxes remains unchanged throughout the range of encountered water potentials. The loss in conductance in the plants is happening between the xylem and the leaf (Eq. S8). Dropping conductances for water fluxes from plant water storage Q_cap_ and root surface Q_soil_ to the xylem could be implemented with appropriate parameterizations, once they are known, resulting in equations Eq. S2 & S5 becoming similar to Eq. S8, if a “Brooks-Corey-like” parameterization of the loss of conductance was used. However, we refrain from implementing this in the model for two reasons: *i)* ideally, the necessary parameters for a “Brooks-Corey-like” parameterization (h_0_ and τ for the respective fluxes Q_cap_ and Q_soil_, respectively) would either be known from experimental studies or calibrated from data, but we could neither find studies specifically quantifying these parameters, nor do we have enough data to calibrate these parameters without having issues of equifinality of model parameterizations, and *ii)* within the range of water potentials that the plants operate at, soil hydraulic conductance for the water flux from the soil to the root starts to drop at much higher water potentials than those required to drop hydraulic conductances of water fluxes within the plant. Therefore, it is much more likely that transpiration is limited by the loss of conductance in the soil rather than the potential loss of conductance between water already in the plant (water at root surface for Q_soil_ and water in plant storage for Q_cap_) and the xylem.

**References**

**Abdalla M, Ahmed MA, Cai G, Wankmüller F, Schwartz N, Litig O, Javaux M, Carminati A**. **2022**. Stomatal closure during water deficit is controlled by below-ground hydraulics. *Annals of Botany* **129**: 161–170.

**Carminati A, Javaux M**. **2020**. Soil Rather Than Xylem Vulnerability Controls Stomatal Response to Drought. *Trends in Plant Science* **25**: 868–880.

**Meinzer FC, Andrade JL, Goldstein G, Holbrook NM, Cavelier J, Jackson P**. **1997**. Control of transpiration from the upper canopy of a tropical forest: the role of stomatal, boundary layer and hydraulic architecture components. *Plant, Cell & Environment* **20**: 1242–1252.

**Peters RL, Pappas C, Hurley AG, Poyatos R, Flo V, Zweifel R, Goossens W, Steppe K**. **2021**. Assimilate, process and analyse thermal dissipation sap flow data using the TREX r package. *Methods in Ecology and Evolution* **12**: 342–350.

**Wankmüller FJP, Carminati A**. **2022**. Stomatal regulation prevents plants from critical water potentials during drought: Result of a model linking soil–plant hydraulics to abscisic acid dynamics. *Ecohydrology* **15**: e2386.
